# SparkMaster 2: A New Software for Automatic Analysis of Calcium Spark Data

**DOI:** 10.1161/CIRCRESAHA.123.322847

**Published:** 2023-08-09

**Authors:** Jakub Tomek, Madeline Nieves-Cintron, Manuel F. Navedo, Christopher Y. Ko, Donald M. Bers

**Affiliations:** 1 Department of Pharmacology, University of California, Davis School of Medicine; 2 Department of Anatomy, Physiology, and Genetics, University of Oxford

**Keywords:** Arrhythmias, Calcium Cycling/Excitation-Contraction Coupling, Computational Biology, Ventricular Function, Imaging

## Abstract

**Background:**

Calcium (Ca) sparks are elementary units of subcellular Ca release in cardiomyocytes and other cells. Accordingly, Ca spark imaging is an essential tool for understanding physiology and pathophysiology of Ca handling and is used to identify new drugs targeting Ca-related cellular dysfunction (e.g., cardiac arrhythmias). The large volumes of imaging data produced during such experiments require accurate and high-throughput analysis.

**Methods:**

We developed a new software tool SparkMaster 2 (SM2) for the analysis of Ca sparks imaged by confocal line-scan microscopy, combining high accuracy, flexibility, and user-friendliness. SM2 is distributed as a stand-alone application requiring no installation. It can be controlled using a simple-to-use graphical user interface, or using Python scripting.

**Results:**

SM2 is shown to have the following strengths: a) high accuracy at identifying Ca release events, clearly outperforming previous highly successful software SparkMaster; b) multiple types of Ca release events can be identified using SM2: Ca sparks, waves, mini-waves, and long sparks; c) SM2 can accurately split and analyze individual sparks within spark clusters, a capability not handled adequately by prior tools. We demonstrate practical utility of SM2 on two case studies, investigating how Ca levels affect spontaneous Ca release, and how large-scale release events may promote release refractoriness. SM2 is also useful in atrial and smooth muscle myocytes, across different imaging conditions.

**Conclusions:**

SparkMaster 2 is a new, much-improved user-friendly software for accurate high-throughput analysis of line-scan Ca spark imaging data. It is free, easy to use and provides valuable built-in features to facilitate visualization, analysis and interpretation of Ca spark data. It should enhance the quality and throughput of Ca spark and wave analysis across cell types, particularly in the study of arrhythmogenic Ca release events in cardiomyocytes.

## Non-standard Abbreviations and Acronyms

RyR: Ryanodine receptor
SM2: SparkMaster 2
SR: Sarcoplasmic reticulum

## Introduction

Calcium ions (Ca) are essential regulators of a multitude of biological functions in virtually all cell types, including oocyte fertilization ^1^, neurotransmitter release ^2^, muscle contraction ^3^, and generation of the cardiac action potential and contraction ^4^. Intracellular [Ca] ([Ca]i) is tightly regulated both spatially and temporally, and dynamical [Ca]i changes come in many forms. The Ca spark is a brief, spatially localized form of Ca release from intracellular Ca stores, initiated by transient opening of a cluster of ryanodine receptor (RyR) Ca release channels in cardiac myocytes sarcoplasmic reticulum (SR) ^5^. It is considered to be a unitary form of stochastic Ca release and has been studied in several different cell types, but especially cardiac myocytes ^6–8^, human induced pluripotent stem cells ^9^, skeletal muscle ^10,11^, smooth muscle ^12,13^, and neurons ^14,15^

In the cardiac myocyte, Ca is central in excitation-contraction coupling, where Ca ions entering via L-type Ca channels bind to and activate RyRs to provide a large and spatially synchronized SR Ca release that causes contraction ^4^. Ca sparks are typically observed in the resting myocyte or during the diastolic phase between normal periodic beats. Ca sparks tend to be larger and more frequent when either SR Ca load or local [Ca]i is raised, and when RyRs become sensitized and leaky in pathological states ^16^. At low Ca spark frequency and amplitude, sparks are usually isolated within the neighborhood of a single RyR cluster or Ca release unit (~1 μm^3^). However, as Ca spark frequency and amplitude increase, they can activate neighboring RyR clusters and form propagating Ca waves via diffusive Ca-induced Ca-release, which in heart can be highly arrhythmogenic, causing delayed afterdepolarizations. This pathological consequence of SR Ca leak has promoted high-throughput screening efforts to identify small-molecule compounds targeting leaky RyRs to mitigate pathological SR Ca leak ^17–20^. Therefore, there is vital and clinically relevant need to rapidly and accurately quantify Ca sparks and waves over a broad range of Ca release behaviors.

Ca sparks are typically recorded with Ca-sensitive fluorescent indicators in longitudinal or transverse line-scan modes with a conventional confocal microscope (2-6 ms/line), producing time-dependent pixel intensities indicative of local [Ca]i at each point along the line (x- or y-t data). 2D imaging (x-y-t) at this time resolution requires higher speed instrumentation, but is feasible and new 2D-Ca spark analysis tools are available ^21,22^. However, line-scan mode Ca spark imaging remains a key method for spark measurements in the field, offering good signal quality on affordable microscopes at a temporal pixel resolution of ~500 Hz.

Cheng et al. ^6,23^ created an initial IDL-based Ca spark analysis tool that was useful, but lacked an easy-to-use graphical user interface (GUI). SparkMaster ^24^, an ImageJ plugin that provides an accessible and practical GUI, was developed afterwards and became a standard tool in the field, complemented by other recent approaches ^25,26^. While SparkMaster remains remarkably capable, it has several limitations which can limit its utility and often warrant extensive manual post-processing. For example, it cannot easily distinguish larger Ca release events such as waves or mini-waves, which has required work-arounds when Ca sparks and waves appear in the same records. SparkMaster also has limitations in spark detection accuracy and options for customization, which is critical when applied to imaging data collected using different microscopes and with different imaging conditions in labs across the world.

Here, we present SparkMaster 2 (SM2), a new open-source tool for analysis of line-scan Ca spark data that brings multiple crucial advancements over its predecessor, while remaining easy to use. SM2 is freely available as an open-source stand-alone application with a robust GUI, as well as a Python module, enabling its further use in a script-based analysis environment. It achieves a much greater accuracy of spark detection through a new spark-detection algorithm and offers a broader range of features compared to SparkMaster. While the software is more complex than the original SparkMaster, it analyzes data at a faster speed, with further speedup being possible by parallelization, making it ideally suited for relatively high-throughput studies.

Below, we 1) present the main functionality of SM2, 2) demonstrate its applicability to real-world Ca spark/wave analysis problems, using two case studies, 3) show that SM2 outperforms not only SparkMaster but also human annotators in terms of spark detection accuracy, using computer-generated synthetic spark data with a known ground truth, and 4) demonstrate that SM2 can be used to detect and analyze Ca sparks in multiple cell types and across different imaging conditions.

## Methods

SM2 is implemented in Python and is distributed freely as an open-source, stand-alone, runnable GUI software application. We selected Python as the programming language for this task for the following reasons: 1) it does not require ownership of expensive software (in contrast with, e.g., Matlab); 2) it is commonly used for image processing tasks and any further development of SM2 does not require nonstandard framework knowledge (in contrast with ImageJ plugins, which are Java-based and require an understanding of a complex underlying framework); 3) it natively supports script-based analyses, including follow-up visualizations and statistical analyses, which are readily available as different Python libraries; 4) it is sufficiently fast to enable high-throughput analyses to be carried out; 5) it makes it possible to produce a stand-alone runnable application. We did our best to make SM2 an easy-to-use and robust tool. However, we emphasize that we are happy to answer any questions and/or offer additional support via the e-mails provided for the corresponding authors.

We confirm the first author had full access to all the data in the study and takes responsibility for its integrity and the data analysis.

### SM2 detection of calcium release events

The general approach to detection of sparks and other release events is as follows: 1) Source images are preprocessed to reduce spatiotemporal background variations and denoising procedures are applied; 2) Candidate release events are detected based on brightness (in a way similar to the original SparkMaster, but much more sensitive); 3) candidate objects are scored based on their size and brightness and are classified as sparks, long-lasting sparks, waves, or miniwaves. Objects with low overall score are rejected as noise artifacts. 4) Splitting procedures are applied to make sure that clusters of release events are appropriately split into unitary events. 5) Finally, visualizations of object bounding boxes are shown. Visualizations of various intermediate results and variables may be toggled on to give insight into SM2’s decision process. In addition, density maps such as in Figure 4 may also be shown.

A detailed graphical overview of SM2 segmentation process is given in Supplementary Methods, where each step is reviewed visually, demonstrating the work carried out there and discussing the rationale for the chosen approach where relevant. The graphical overview also lists which parameters are used in which step exactly, helping the user understand each parameter’s interpretation.

### SM2 analysis of spark properties

For each detected object, a number of features is extracted, including all typically reported spark properties such as amplitude, full duration and width (both at full- and half-amplitude), tau of decay, number of sparks and other events in a recording, spark frequency (per 100 μm per s). The outputs are saved as a csv spreadsheet, facilitating further statistical analysis. A summary spreadsheet is also produced (with a single line per file analyzed), giving median and interquartile range for each feature.

To obtain the trace of a spark fluorescence over time, the sub-image containing the spark is averaged over space, ignoring pixels belonging to other release events (edges of which may be present in the processed sub-image).

### Synthetic data generation

We aimed to generate reasonably plausible data with appropriately shaped sparks, which show certain patterns that were relatively common in our real-world data, such as background variation, couples of adjacent sparks, or repetitive sparks, where an image column contains a repetition of a relatively similar spark, which is present at the same spatial location. No larger release events than single sparks were generated to keep the analysis of the synthetic data focused on spark detection, which will be the main task of SM2. Examples of produced data are in Figure 5. The procedure overview is given below (the Matlab code is uploaded to the project’s Github). Most parameters are randomized within pre-selected bounds and a large number of images can be created rapidly. The parameter bounds were set so that the sparks produced are consistent with spark properties in the real recordings (i.e., unrealistically small or large sparks are not possible) We note that the range of brightness levels of added sparks was chosen to make the dimmest sparks difficult or impossible to see, as this enables a comparison of differential sensitivity of various tools. If the data contained only clearly visible sparks, these would be all detected by reasonably capable detection systems, not allowing measurement of differences in the rate of false negatives.

1. A “library” of multiple reference spark shapes was built using real spark recordings. When a spark is added to the recording, it is randomly picked from this library.
2. Background image is created, initially as a blank slate with random intensity within a certain range. To this are added a number of dark or bright bands (random shift in brightness vs background), mimicking the background variation in real data. Finally, each column is multiplied by a random number close to 1 to add further spatial variation.
3. 0-2 repetitive spark bands are added (these are columns with a – not necessarily periodically – repeating spark). Before being repeated and embedded in the background, a random reference spark is rescaled randomly in both dimensions, and its intensity is randomly varied.
4. Multiple spark couplets are added. These are pairs of sparks (with random scaling and randomized brightness) that are adjacent or near-adjacent. One spark is placed first, with the second one being placed at a random angle to it and at a random distance (within a relatively tight interval, ensuring the spark adjacency).
5. A large number of sparks (with randomized scaling and brightness) are added to the data, so that they are not too close to any other spark (to prevent unrealistic overlapping).
6. Gaussian noise is added (with standard deviation randomly selected within a given range) to the whole image.
7. Images are stored, as well as reference masks containing segmentation of true spark locations, useful for later analysis.

### Comparison of SM2 with SparkMaster and human annotators on synthetic data

In order to compare the accuracy of spark detection of SM2 versus SparkMaster and human

annotators, we designed the following evaluation:

1. 30 synthetic images with Ca sparks (of various numbers, typically between 30 and 100 per image), created as described in the previous section, including the ground truth annotation of where the sparks are located. A total of 1561 sparks were present in this dataset.
2. Each of these images is annotated using SM2 and SparkMaster, generating a segmentation mask of predicted spark locations. SparkMaster produces only bounding boxes of predicted release events, so in order to produce a similar shape of segmented objects as SM2 or humans, an ellipse inscribed into the bounding box rectangle was used.
3. Six human annotators annotated the 30 images in a way that every image was annotated by exactly two humans, enabling the calculation of inter-annotator agreement.
4. For each system (SM2, SparkMaster, and humans), the predicted spark locations were compared to the ground truth, using a graph-based approach to count true positive, false positive, and false negative spark detections in the following way:
  - A bipartite graph was constructed with the partite vertices corresponding to the ground truth and predicted objects respectively. An edge is placed between two vertices when the Dice coefficient between the corresponding ground truth and predicted object is at least 0.15. The Dice coefficient is defined as $\frac{2|GT\cap P|}{\left|GT|+|P\right|},$ where |*GT*| is the number of pixels of the ground truth object, |*P*| is the number of pixels of the predicted object, and |G ∩ T| is the number of pixels that the ground truth and predicted object share. I.e., for a full overlap between true and predicted object, the Dice coefficient is 1, and when the two object do not overlap, it is zero. In this way, the edges in the graph correspond to a “reasonable” overlap between the ground truth and prediction.
  - A maximum bipartite matching is found and:
    - Ground truth vertices with no outgoing edge correspond to false negatives (a true object that was not found).
    - Predicted vertices with no outgoing edge correspond to false positives (a predicted object not overlapping with a true object).
      An advantage of this approach is that it enables detecting errors due to over-segmentation and/or under-segmentation of spark clusters, which cannot be resolved merely by looking at whether a predicted object has an overlap with a true one and vice versa. For example, two nearby true sparks that are segmented as a single predicted object containing both will yield the scenario when two ground-truth-partite vertices are connected by an edge to one predicted-partite vertex. In the maximum bipartite matching, one of the edges is selected, leaving one unmatched ground-truth partite vertex, contributing a single false negative error.
5. False positive and false negatives are counted and compared across the three systems.

SM2 performance was compared to that of SparkMaster, given its gold standard status in the field. In addition, it was not possible for us to analyze the data using other tools, due to their lack of documentation and/or crashes.

### Experimental conditions

#### Mouse ventricular myocyte isolation and Ca spark imaging data used in Figures 1 – 5

This dataset was collected during the course of a previous study ^29^. There, ventricular myocytes were enzymatically isolated (300 U/ml collagenase Type II, Worthington; ≥ 9.8 U/ml protease Type XIV, Sigma) by retrograde Langendorff perfusion (37°C) from male C57Bl/6J mice (6-8 weeks old). All procedures complied with the policies of the Animal Research Committee of the University of California, Los Angeles. Spontaneous Ca activity (from sparks to waves) were evoked in saponin-permeabilized myocytes (0.005% w/v, 30-60 s) by varying free [Ca] in mock internal solution composed of (in mmol/L) 100 potassium aspartate, 20 KCl, 5 KH_2_PO_4_, 5 MgATP, 10 phosphocreatine, 5 U/ml creatine phosphokinase, 10 HEPES, 0.25–1 EGTA, 1 MgCl_2_ (free), 0.03 Fluo-4 (Invitrogen), 50-500 nmol/L CaCl2 (free; calculated by MaxChelator), and 8% w/v dextran ^30^, pH 7.2 (KOH). Ca fluorescence was recorded (Ex: 488 nm; Em: >510 nm) using a Zeiss PASCAL 5 laser scanning confocal system (Carl Zeiss) on a Zeiss Axiovert 100 LSM inverted microscope fitted with a 63X objective (Zeiss C-Apochromat 63/1.2 W Corr) in the line-scan mode (1.92 ms/line, 2604 lines/recording) along the longitudinal axis and digitized into 1024 × 2604-pixel (12-bit) images with nominal pixel width of 0.08–0.13 μm.

#### Rabbit Atrial Myocyte Isolation and Ca spark recording

Atrial myocytes were isolated from New Zealand White rabbits (male, 3- to 4-month-old, Charles River Laboratories) using a standardized enzymatic technique as previously described ^31^ and approved by the University of California, Davis Institutional Animal Care and Use Committee. Briefly, animals were injected with heparin (400 U/kg body weight) and subjected to general anesthesia induced via intravenous injection of 10 mg/kg body weight propofol (Rapanofal®, Ivaoes Animal Health, Miami, FL, USA) followed by 2–5% isoflurane inhalation in 100% oxygen throughout the procedure. Deep surgical anesthesia was confirmed by abolished pain reflexes. All animals were euthanized by surgical excision of the heart while in deep anesthesia. Immediately after excision, the heart was rinsed in cold nominally Ca^2+^-free modified Tyrode’s solution composed of (in mmol/L): NaCl 135, KCl 5.31, MgCl_2_ 1, HEPES free acid 10, Na-HEPES 10, NaH_2_PO_4_ 0.33, Na pyruvate 2, glucose 5.5; pH 7.4. The aorta was cannulated and retrogradely perfused on a constant flow Langendorff apparatus at 37°C with modified Tyrode’s solution with (in mmol/L) 0.02 CaCl_2_ and taurine 8, and gassed with 100% O_2_. Collagenase Type II (Worthington Biochemical Co., Lakewood, NJ, USA) and Protease Type XIV (Sigma-Aldrich) were used for enzymatic digestion. Atrial myocytes were mechanically dissociated, filtered through a nylon mesh, and allowed to sediment for 10 min. Sedimentation was repeated three times with modified Tyrode’s solution in which [Ca^2+^] was incrementally increased from 0.01 to 0.025 mmol/L and bovine serum albumin was decreased incrementally from 2% to 0%. Atrial myocytes were kept at room temperature until use. All animal handling and laboratory procedures were in accordance with approved protocols (#23175) of the Institutional Animal Care and Use Committee at University of California, Davis conforming to the NIH Guide for the Care and Use of Laboratory Animals (8th edition, 2011).

To measure [Ca]_i_, isolated myocytes were incubated with the Ca-sensitive fluorescent indicator, Fluo-4 AM (10 μmol/L, Invitrogen) with Pluronic F-127 (0.02%, Invitrogen) in modified Tyrode’s solution ([Ca] = 0.025 mmol/L) for 30 min at room temperature followed by 3 washes and de-esterification for 30 min using Normal Tyrode’s solution composed of (in mmol/L): NaCl 140, CaCl_2_ 1.8, MgCl_2_ 1, KCl 4, Na-HEPES 5, HEPES free acid 5, Glucose 5.5; pH 7.4. Ca fluorescence was recorded (Ex: 488 nm; Em: 500-550 nm) using a Nikon Eclipse *Ti* laser scanning confocal system (40X objective (water correction)) in the line-scan mode (2 ms/line) along the longitudinal axis and digitized into 512 × 7,500 pixel images (12-bit) with nominal spatial resolution of 0.223 μm/pixel, respectively. Intact myocytes were paced at 1 Hz by field stimulation in Normal Tyrode’s solution ([Ca] = 3.6 mmol/L).

#### Vascular Smooth Muscle Cell Isolation and Ca spark recording

Vascular smooth muscle cells were obtained from mesenteric arteries of male C57Bl/6J mice and cerebral arteries of male GCaMP2 mice (Jackson Labs). Mice were euthanized by intraperitoneal injection of sodium pentobarbital (250 mg/kg), as approved by the University of California, Davis Institutional Animal Care and Use Committee. Cerebral and mesenteric arteries were dissected in ice-cold dissection buffer composed of (in mM): 140 NaCl, 5 KCl, 2 MgCl_2_, 2 10 D-glucose, and 10 HEPES, pH 7.4 with NaOH. Following dissection, arteries were cut into small pieces and digested in dissection buffer supplemented with papain (26 U/ml) and dithiothreitol (1 mg/mL) at 37°C for 7 min for cerebral arteries and 9 min for mesenteric arteries. Following this, arteries were incubated in a dissection buffer containing 0.3 mg/ml collagenase type H and 0.7 mg/ml collagenase type F for 7 minutes at 37° C for cerebral arteries, and 1.77 mg/mL of collagenase type H, 0.5 mg/mL elastase and 1 mg/mL trypsin for 9 minutes at 37° C for mesenteric arteries. After digestion, arteries were washed three times in ice-cold dissection buffer followed by two additional washes in a buffer containing (in mM): 125 NaCl, 5.4 KCl, 15.4 NaHCO_3_, 0.33 Na_2_HPO_4_, 0.44 KH_2_PO_4_, 3 Sucrose, 10 D-Glucose and 11 HEPES, pH 7.4 with NaOH and supplemented with 50 nM CaCl_2_. Vascular smooth muscle cells were placed in a 200 μL recording chamber for imaging.

Cells were bathed with an experimental solution containing (in mM): 140 NaCl, 5 KCl, 1 MgCl_2_, 2 CaCl_2_, 10 D-Glucose, 10 HEPES, pH 7.4 with NaOH. Ca sparks were imaged in vascular smooth muscle loaded with the Ca-sensitive fluorescent indicator Cal 520-AM (5 μmol/L), Fluo 4-AM (5 μmol/L), or from the GCaMP2 expressing mouse using an Andor spinning disk confocal microscopy system coupled to an Olympus iX81 inverted microscope equipped with a 60x water immersion lens. Images were acquired at 90-110 Hz using the Andor IQ software.

### Generation of pseudo-line-scans from 2D spark imaging data

The steps below can be used to generate line-scans from videos of Ca sparks in vascular smooth muscle cells, using Fiji ^27^:

1. A tif stack with the recording is loaded in Fiji.
2. Denoising is applied using Process/Filters/Gaussian blur. We used the value of 1 for the sigma parameter, subsequently confirming the filtering is to be applied to all frames.
3. Additional smoothing is carried out by Process/Smooth.
4. The line tool is selected (on the main Fiji panel, below the menus); right-clicking the button enables the user to select whether a straight or segmented line will be drawn. For smooth muscle myocytes that are typically curved, the segmented line helps to create a pseudo-line scan that is analogous to longitudinal line scans in the more orthogonally shaped ventricular myocytes.
5. Background subtraction is performed by Process/Subtract Background, using a rolling ball radius of 25 pixels, making sure that the checkbox “Light background” is not checked.
6. With the line drawn, a line-scan can be generated via Image/Stacks/Reslice (using the default parameters).
7. A line-scan image is opened and can be saved via File/Save as.

We note that using the segmented line has the advantage of being able to capture more cellular locations within the generated line-scan, and obtain more sparks in the recording than when using a straight line. However, care must be taken to not have segments that pass a single spark or wave (overrepresenting that event), and be consistent in segmenting among cells that are to be compared (full cell length, active zone(s), similar number of linear segments), to minimize biasing interpretations.

## Results

### Demonstration of main functionality of SM2

**Figure 1:**
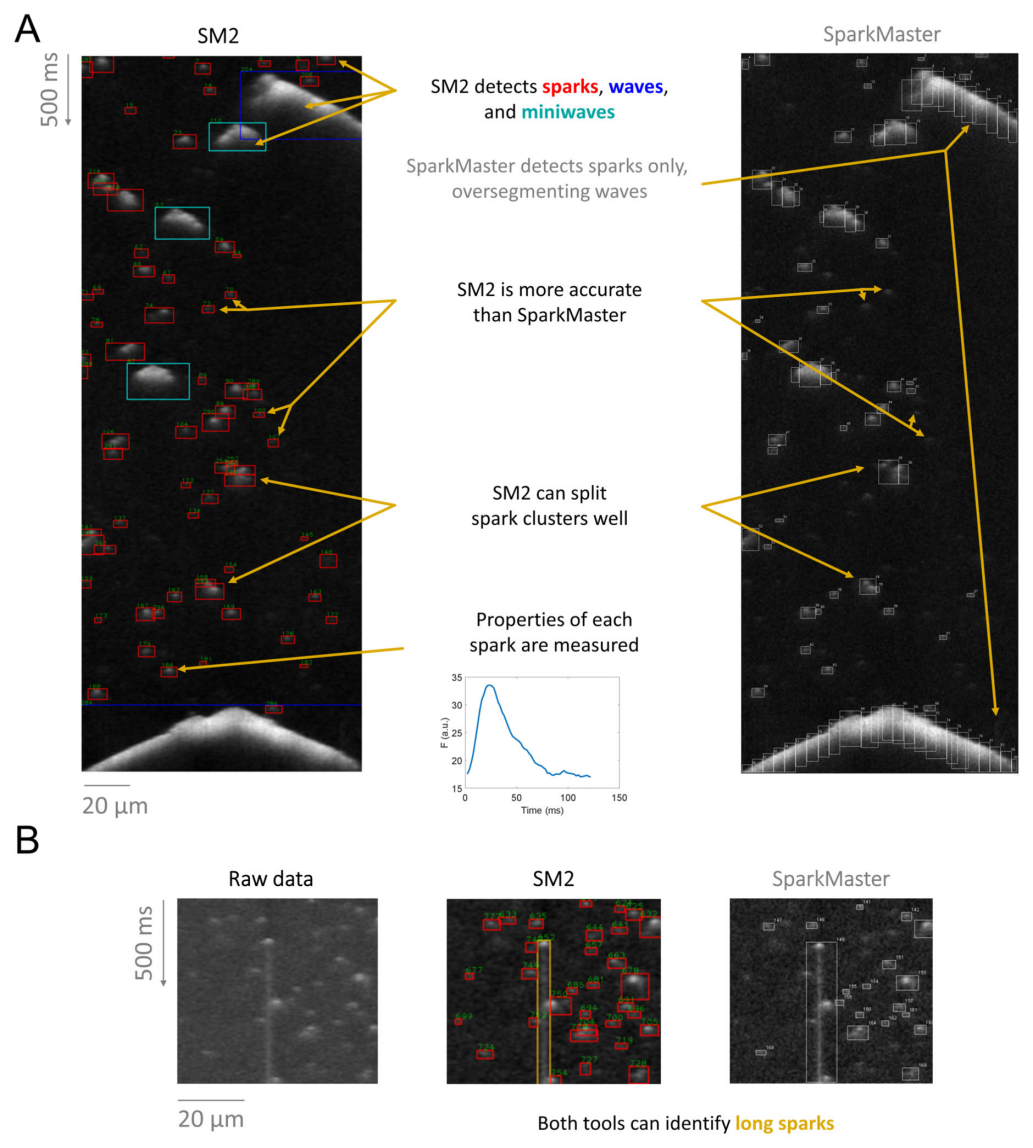
Demonstration of main features of SM2. **A)** Sample segmentation of a recording using SM2 (left) versus the original SparkMaster (right). **B)** An illustration of long spark detection by both SM2 and SparkMaster.

Detection and segmentation of Ca release events in experimental data using SM2 has a number of novel features that set it apart from previous approaches such as SparkMaster. Figure 1A compares analysis of a complex record using SM2 vs. SparkMaster. SM2 robustly detects Ca sparks but can now also accurately detect and identify other Ca release events, such as miniwaves and waves (that are indicated in blue and cyan boxes). In the case of these larger release events, SparkMaster would over-segment them into pseudo-sparks, potentially misestimating the spark count and perturbing summary statistics of single-spark properties. Capturing characteristics of these larger events provides an opportunity to use them toward an integrated SR Ca leak rate in records containing multiple event types.

Second, SM2 is substantially more accurate than SparkMaster in detecting Ca sparks, attributed to our new spark detection algorithm (discussed in detail in the last section of Results). SM2’s algorithm utilizes both brightness and size of detected objects to identify release events, unlike SparkMaster, which uses only the brightness. In particular, relatively dim sparks can be detected by SM2, whereas such sparks would be either missed by SparkMaster, or would require such a low fluorescence detection threshold that their detection would lead to a substantial number of false positive sparks.

Third, SM2 contains dedicated functionality for splitting clusters of Ca sparks, identifying underlying isolated sparks. In contrast, SparkMaster often labels spark clusters as one object, or splits them incorrectly. Finally, in certain experimental conditions, so-called “long sparks” may emerge ^24^, lasting for hundreds of milliseconds. Both SM2 and SparkMaster can identify the long sparks (Figure 1B), but enabling the detection of long sparks (by a toggle) is highly computationally demanding in SparkMaster (increasing analysis time to several minutes per single image); conversely, it makes SM2 analysis only marginally more time-consuming (less than a second of additional runtime per image).

**Figure 2:**
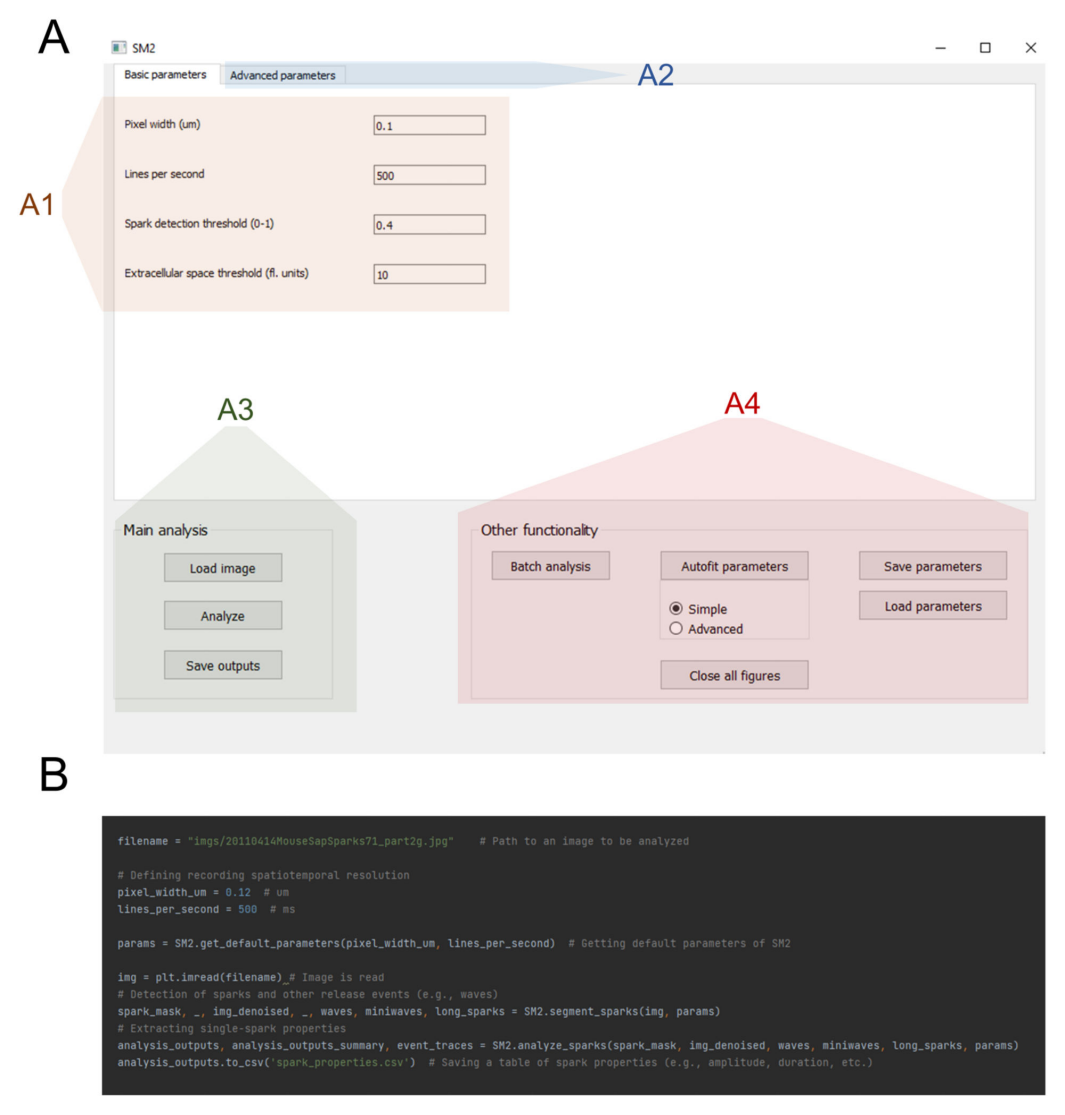
SM 2 controls. SM2 may be controlled either using a graphical user interface **(A)** or by writing Python scripts **(B).** The GUI provides access to setting of basic parameters (A1), advanced parameters changing details of analysis procedures (A2), single-recording analysis (A3), and other functions, including batch processing and parameter saving/loading (A4). The color overlays are added only for the purpose of this figure and are not present in the actual software.

SM2 can be primarily controlled using a GUI (Figure 2A) and is distributed as a stand-alone application, not requiring specialist knowledge or installation to run. The GUI allows setting of basic analysis parameters (Figure 2A1), which are mostly identical to those of SparkMaster, facilitating the transition from SparkMaster to SM2. At the same time, advanced parameters may be tweaked by advanced users to allow additional control over the detection of release events and their analysis (Figure 2A2). This includes the possibility to visualize intermediate results of the analysis, giving insight into how exactly the software works during the analysis and which parameters are to be changed to alter its behavior to the operator’s preference. Offering such intermediate visualization partly overcomes the “black box” perception of some analysis tools, allowing improved ability to diagnose and remedy any unexpected results from the software.

The analysis in the GUI is carried out by loading an image to be analyzed, carrying out the automated analysis, with the results being stored as csv spreadsheets (Figure 2A3). Analysis outputs contain many features for each event, such as spatiotemporal dimensions of the release events, tau of [Ca]_i_ decay, and all the other measured features in the original SparkMaster, as well as summary statistics of the features across the recording. Such automated analysis is easily complemented by visual inspection of the spark segmentation (shown, e.g., in Figure 1), which is an important step in quality control, and is displayed by the software by default.

Other functionality accessible through the GUI includes batch file analysis, saving/loading parameter sets, or parameter “autofitting” (Figure 2A4). The autofitting provides ways of searching through the parameter space so that the segmentation of Ca release events best corresponds to user-provided reference annotations. In this way, the behavior of SM2 can be optimized not only for maximum accuracy, but e.g. also for searching only for very clear and/or large release events, depending on how the user-provided reference annotation is created.

In addition to the GUI control of SM2, it can also be controlled via Python scripting (Figure 2B), using SM2 as a module. This enables an even greater degree of automation than the batch analysis provided in the GUI, also facilitating the integration of SM2 into other analysis pipelines as an intermediate analysis tool. Finally, script-based control makes it easy to follow an image analysis with a subsequent statistical analysis and/or visualization in the same environment, which is beneficial for research reproducibility and tractability.

### Case study 1: Calcium release events change with increasing [Ca]

**Figure 3:**
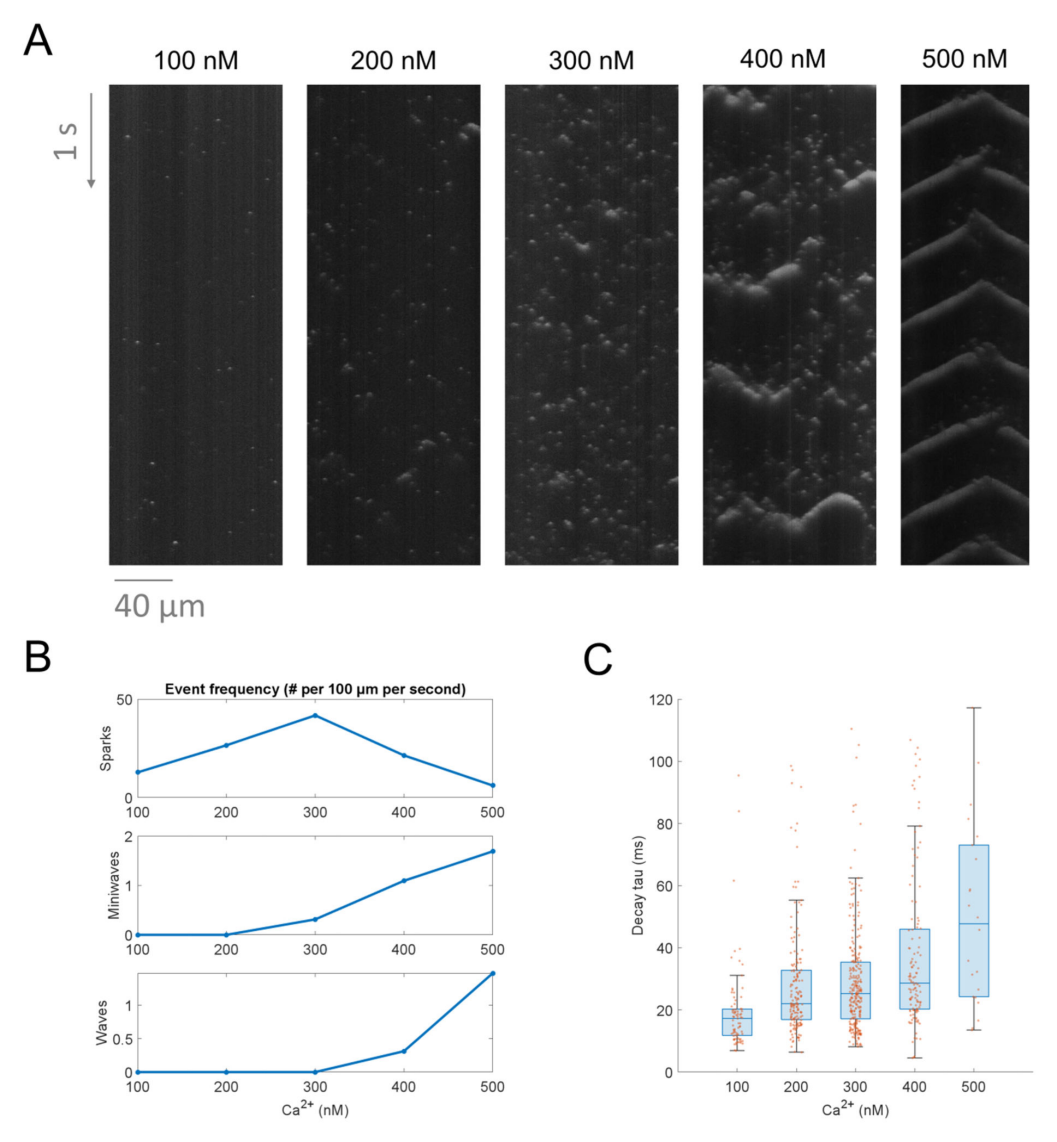
Using SM2 to analyze the effect of increasing [Ca]_i_, on Ca release events. **A)** A comparison of the Ca release patterns in five distinct permeabilized cells exposed to a variety of calcium concentrations. **B)** A summary of frequencies of Ca sparks, miniwaves, and waves, in the five recordings above. **C)** A comparison of calcium spark decay time constants across the different concentrations (using standard boxplots in blue and the underlying data points in orange). To focus this analysis on sparks only, Ca miniwaves and waves were excluded, as well as sparks with decay time constant over 120 ms, indicating an incorrectly segmented object and/or failure of the time constant fitting procedure

A typical use case of SM2 will be to understand how exposure to different conditions affects the pattern of Ca release events and their properties. As a proof of concept, we analyzed data acquired in permeabilized murine ventricular myocytes exposed to multiple known [Ca]_i_, with the permeabilization allowing direct control over the steady state [Ca]_i_ in the intracellular space. The resulting spontaneous Ca release events were recorded using the fluorescent Ca indicator Fluo-4 (Figure 3A). It can be seen that Ca spark frequency peaks at 300 nM [Ca]_i_, whereas the frequency of Ca miniwaves and waves increases monotonically with the [Ca]_i_ (Figure 3B). Taken together, the data may be interpreted in the following way: 1) increasing [Ca]_i_ promotes spontaneous Ca release, as evidenced by the increase in spark frequency between 100 and 300 nM. 2) However, increasing the [Ca]_i_ further leads to such a degree of Ca release enhancement that the sparks start organizing into increasingly more synchronized Ca release patterns, from macro-sparks (that may involve only 2-4 release sites) to more mini-waves and waves, but resulting in a reduced frequency of detected Ca sparks.

Beyond comparing recording-wide properties such as spark frequency, SM2 outputs may be used to compare single-spark properties across different experimental conditions. For example, Figure 3C shows the comparison of Ca spark tau (time constant of decay) across the five tested [Ca]_i_. This indicates that the higher the [Ca]_i_ is, the longer is the decay time of Ca sparks, which is consistent with the overall facilitation of Ca release by elevated [Ca]_i_ (which can depend on both the local cytosolic [Ca]_i_ as well as the luminal SR [Ca] ^16^.

### Case study 2: Using SM2 outputs to observe refractoriness of SR Ca release

**Figure 4 F4:**
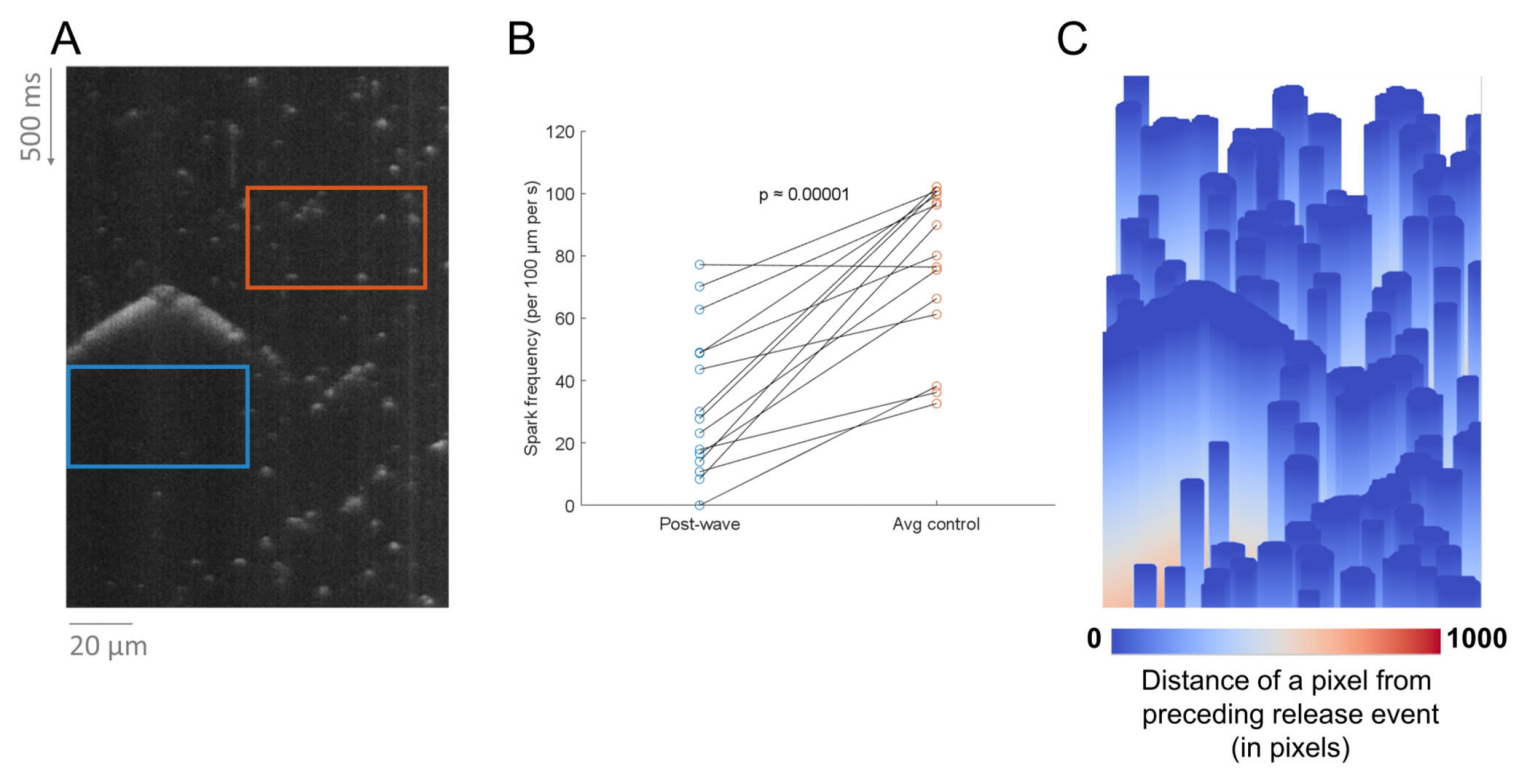
Comparing spark frequency following large release events versus other areas. A) An illustration of a box lasting 150 ms following a wave (blue) and a same-size box in a randomly selected position within the recording (orange). B) The difference.

In addition to comparing properties of recordings taken in different conditions, SM2 can be used also to analyze differential properties of Ca release events within recordings. For example, we used it to test the hypothesis that large Ca release events (e.g. waves) could delay the appearance of additional Ca sparks, corresponding to either local RyR refractoriness or reduced local luminal [Ca] inside the SR (Figure 4A, B), both of which may temporarily reduce RyR open probability and Ca spark frequency. Indeed, we see that the Ca spark frequency in the time window of 150 ms following a wave or mini-wave is markedly lower than in a random region in the same recording not preceded by waves (*p* ≈ 0.00001, paired t-test). Importantly, this behaviour was consistent across waves in different areas of the cells, meaning that the difference does not reflect the blue boxes merely being located in zones with *a priori* low spark rate (which would be furthermore hard to reconcile with the emergence of waves).

SM2 also contains functionality for visual illustration of spark density, such as shown in Figure 4C, where the distance of each pixel from a preceding release event is shown for the image in Figure 4A. The warm-color regions of the image correspond to areas that are late after a preceding release event. In this case, the warm-color areas are present following the Ca wave but not typically following sparks, illustrating the SR release refractoriness (or depletion) following a large release event. We anticipate that in the future, analyses such as this may be also performed in the setting of comparing multiple types of experimental recordings, for example quantifying the rate of recovery from refractoriness in different experimental conditions.[Fig F4]

### SM2 outperforms SparkMaster and human annotators on synthetic spark data

**Figure 5 F5:**
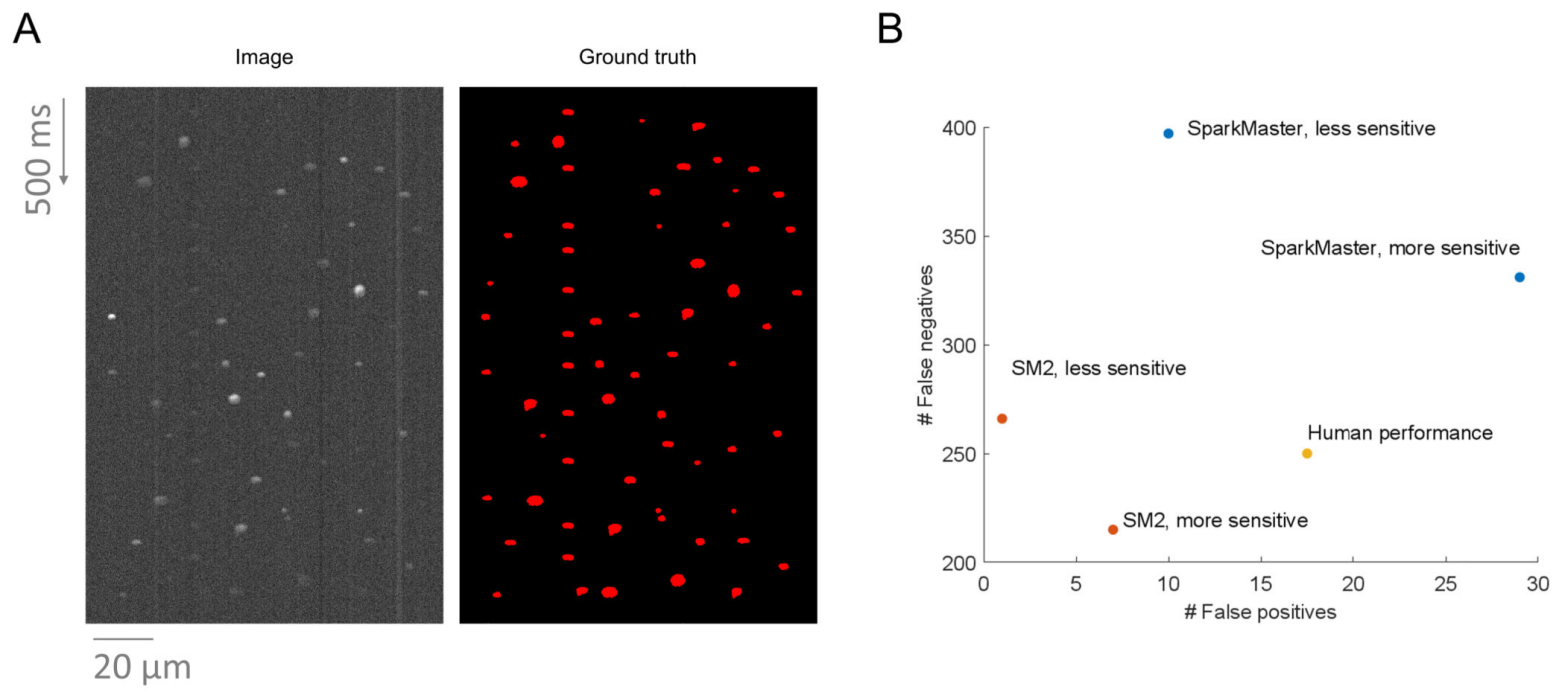
Comparison of SM2, SparkMaster, and human annotators using synthetic data. **A)** An example of synthetic data produced, with a synthetic image to the left and a mask of ground truth spark segmentation to the right. **B)** The comparison of the performance of the three annotation systems. SparkMaster used thresholds of 3.4 and 3.8 for the more and less sensitive version respectively. SM2 used the thresholds of 0.45 and 0.6 for the more and less sensitive annotation (the threshold values are not comparable between SparkMaster and SM2). The total number of false negatives and false positives across 30 images in the dataset is shown.

To assess the spark detection performance of SM2, we built a dataset of synthetic line-scan images of Ca sparks. Using synthetic data has the intrinsic advantage that the ground truth is known, and this enables accurate quantification of detection errors. The spark data produced (example in Figure 5A) were based on existing spark morphologies and include phenomena such as uneven background intensity, couplets of very near sparks, or occasional presence of a repeated spark in a single spatial location (details provided in Methods). In addition to SM2, we also measured the spark detection accuracy of SparkMaster, as well as of six human volunteers. Thirty images were used for this study, with each human annotating ten images in total; the files were distributed to annotators in a way where each image has been annotated by two different persons. In the case of software tools, two different spark detection thresholds were investigated, corresponding to more and less sensitive detection.

Strikingly, the default spark detection using SM2 (more sensitive) is not only markedly more accurate than the original SparkMaster (even at its more sensitive setting), but it also surpasses the average of human annotators, yielding both fewer false positives and false negatives (Figure 5B, examples in Supplementary File 1). The less sensitive parametrization of SM2 leads to only marginally more false negatives compared to human, but at the same time it yields far fewer false positives. While these highly encouraging results may not be universal across all types of real-world data, they nevertheless indicate a very strong spark detection performance of SM2. We carried out an additional analysis of the relatively weak performance of SparkMaster (Supplementary File 2), observing that the central problem is the poor segmentation of dim Ca sparks by SparkMaster. This issue can manifest both as false positive and false negative detection (discussed in Methods), and while it is not a problem when estimating relative spark rate using SparkMaster, it is likely to substantially perturb the estimation of spark features.

Finally, we calculated the inter-annotator agreement among the human annotators. For each person, we calculated the total number of true positives, false positives, and false negatives, using the other persons’ annotations as ground truth reference. Subsequently, the agreement of an annotator with other annotators was defined as $\frac{\#\;true\;positives}{\#\;true\;positives\;+\;\#\;false\;positives\;+\;\#\;false\;negatives}$.

The average of this score across annotators was 0.896 with the standard deviation of 0.023, indicating an overall strong agreement. [Fig F5]

### Demonstration of SM2 generality

**Figure 6:**
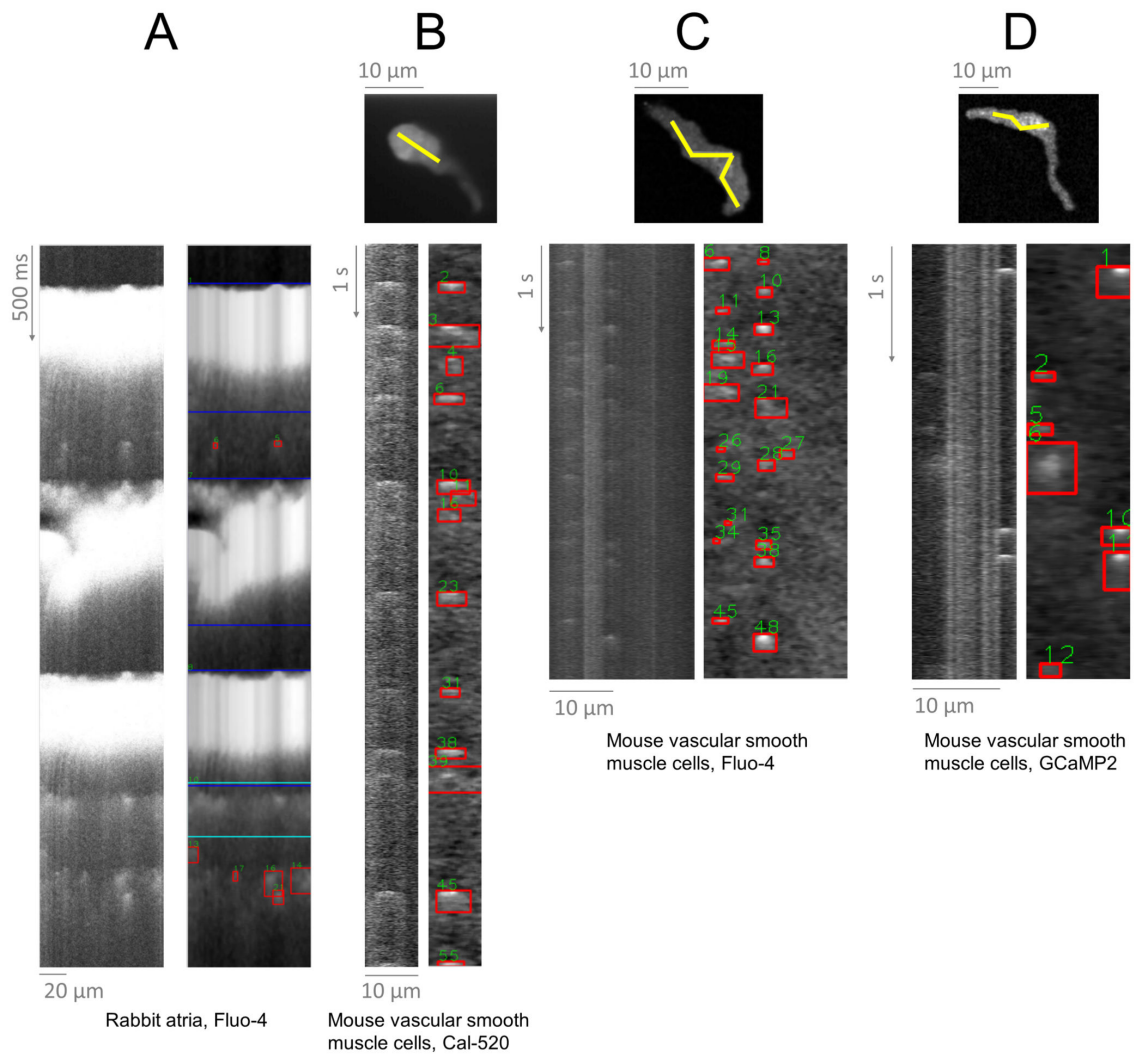
Application of SM2 to distinct cell types and conditions. **A)** Paced rabbit atrial cells loaded with Fluo-4. **B)** Mouse vascular smooth muscle cells imaged using a spinning disk confocal microscope and the Ca dye of Cal-520. Top image shows recorded images, with yellow line being used to extract the line-scan recording underneath. **C, D)** Analogous recordings of vascular smooth muscle cells, using Fluo-4 and GcaMP2 respectively to image the Ca sparks. Performance of the original SparkMaster on these recordings is shown and discussed in Supplementary File 3.

Prior sections demonstrated the utility of SM2 in analyzing Ca sparks in mouse ventricular myocytes. However, SM2 is broadly applicable for Ca spark and wave analysis. Figure 6A demonstrates spark detection in intact paced rabbit atrial myocytes (non-permeabilized). Analyzing sparks within paced recordings is something that the original SparkMaster does not handle adequately (Supplementary File 3), but SM2 performs well. SM2 can also be also used to detect and analyze sparks in intact mouse vascular smooth muscle cells (Figure 6B-D), which are typically non-linear in morphology and smaller when compared to cardiomyocytes.

Data in Figure 6B-D were collected using a spinning disk microscope, which records a video of a cell, rather than a line-scan (i.e., recording a x-y plane repeatedly in time, rather than a line). While such data cannot be directly processed by SM2, they can be easily converted into line-scans using the free tool Fiji ^27^ along user-defined line (see Methods), with resultant line-scans subsequently loaded into SM2. Sparks in thus-generated line-scans can readily be detected by SM2, demonstrating utility beyond cardiac research. Moreover, recordings in Figure 6B-D were collected using two small molecule Ca indicators (Cal-520 and Fluo-4), or a genetically encoded Ca sensor (GCaMP2), showing utility across fluorophores. In summary, SM2 can be used to analyze Ca sparks across cell types and imaging conditions and sensors.

## Discussion

The Ca spark is a RyR-mediated subcellular Ca release event which continues to serve as an important functional indicator of intracellular Ca activity in numerous physiological systems. There is also a growing interest in the study of Ca sparks in high-throughput screening strategies to identify small-molecule compounds that target RyRs to therapeutically mitigate pathological SR Ca release ^17–20^. The ability to quickly and accurately detect, analyze, and quantify Ca sparks, therefore, remains essential for gaining mechanistic insights on Ca-mediated biological processes. Recent novel findings and key unresolved questions in the field of Ca-related studies have highlighted the need for advancements in analytical tools and approaches.

Here, we present SparkMaster 2 (SM2) as a next-generation all-in-one software-based tool for high-throughput analysis of Ca spark data acquired in the line-scan mode. Its key strengths are: 1) much improved accuracy of identification of Ca sparks, resulting from a more sophisticated detection algorithm, more extensive image pre-processing, and a custom algorithm to separate spark clusters into single sparks; 2) the capability to identify more complex Ca release events, such as Ca waves or miniwaves, which makes possible the robust analysis of recordings exhibiting a complex and dynamic mix of distinct types of release events; 3) it reports all commonly reported properties of Ca sparks, as well as novel features, such as spark latency, enabling the investigation of local Ca release refractoriness; 4) it can be controlled either via a convenient graphical user interface (distributed as a stand-alone application) or in a Python script-based analysis environment.

Practical utility of SM2 is demonstrated in this article using case studies based on real-world data, also showing broad applicability to analysis of Ca sparks in multiple cell types imaged in distinct conditions. The accuracy of SM2 spark detection is more formally demonstrated using synthetically generated spark data (Figure 5), where SM2 outperforms the original SparkMaster software and, notably, human annotators as well. This means, together with other advantages of SM2, that the software can be used with much less post-processing and human correction than its predecessor. Taken together, our results indicate that SM2 is mature enough to enable automated analysis of Ca spark properties in high-throughput screening studies, as well as new studies in basic cellular cardiology.

Importantly, SM2 was designed with the user experience and versatility of use in mind. Accordingly, the input parameters in the graphical user interface have remained largely consistent with that of the original SparkMaster software to maintain familiarity. In addition, while the default Ca spark detection parameters perform well in common linescan image acquisition conditions, they can be easily customized to accommodate more specialized conditions. To aid this process, the software can visualize intermediate calculation steps graphically, giving insight into its decision-making, and highlighting which parameters need to be changed and how. To further aid users in understanding how SM2 works and how to adapt its behavior, we present the main underlying methodology graphically (Supplementary Methods), rather than relying purely on text or pseudocode. In this way, readers interested in how SM2 works may immediately see what happens in which processing step, rather than having to imagine and infer this.

In recent years, machine learning and artificial intelligence-based strategies have been implemented into many image processing software algorithms as a way to overcome technical difficulties inherent in traditional methods of object detection and to meet or surpass the accuracy of human performance on a high-throughput scale. These strategies were considered for implementation in SM2, and, in fact, the earliest iterations of SM2 were built almost entirely on machine learning strategies. Ultimately, we opted to forego this direction of development for the time being, due to a number of significant limitations. The main one is the lack of large volumes of training data, even for fine-tuning a pre-trained network such as Detection Transformer ^28^ that we attempted, and the high human-hour cost of generating them. Second, generating training data implies an inherent performance ceiling determined by the method of annotation (such as by human, or by SparkMaster). Third, there is a high risk that a machine learning approach based on a training dataset acquired in particular conditions might work poorly on different data and conditions from other labs, limiting the generality of such an approach. Finally, we were concerned by peculiar errors made by the development versions of the neural network. While these did not perform disastrously, they sometimes failed to detect completely obvious Ca sparks that no human operator would miss. While machine learning strategies still hold great promise for eventually advancing image processing performance beyond what is currently possible, the version of SM2 that we present here performed far superiorly to that of our machine learning-based prototype designs.

In conclusion, we anticipate that SM2 will prove to be a valuable tool and will help to resolve fundamental and emerging questions in studies involving Ca sparks and a wider range of complex intracellular Ca activity.

## Supplementary Material

ARRIVE Guidelines

Data Supplement

Major Resource Table

Supplementary File 1

Supplementary File 2

Supplementary Material 3

## Data Availability

Please visit https://github.com/jtmff/SparkMaster2 to download the SM2 software app (for Windows, Mac, or Linux), sample test images, and a User guide, or to find the source code.

## Novelty and Significance

### What is known

- Calcium is a central mediator of muscle contraction, and of many subcellular signaling pathways.
- Calcium handling dysfunction contribute to arrhythmias and other disease phenotypes, and can be an attractive target for treatment.
- Imaging of unitary calcium release events (calcium sparks and waves) via confocal or microscopy is a crucial research technique, but generates large data sets that require quantitative software analysis.
