# Supplementary material for: SparkMaster 2: A New Software for Automatic Analysis of Calcium Spark Data
Source: Circ Res. 2023 Aug 9;133(6):450–62. doi: 10.1161/CIRCRESAHA.123.322847 (PMC7615009; doi:10.1161/CIRCRESAHA.123.322847)
Supplement: Supplementary file 1 [file res-133-450-s001.pdf]

Supplementary material 1

The two following slides contain examples of spark detection on two synthetic images by a human, SM2, and SparkMaster.

Here, thresholds used were 0.45 for SM2 and 3.8 for SparkMaster.

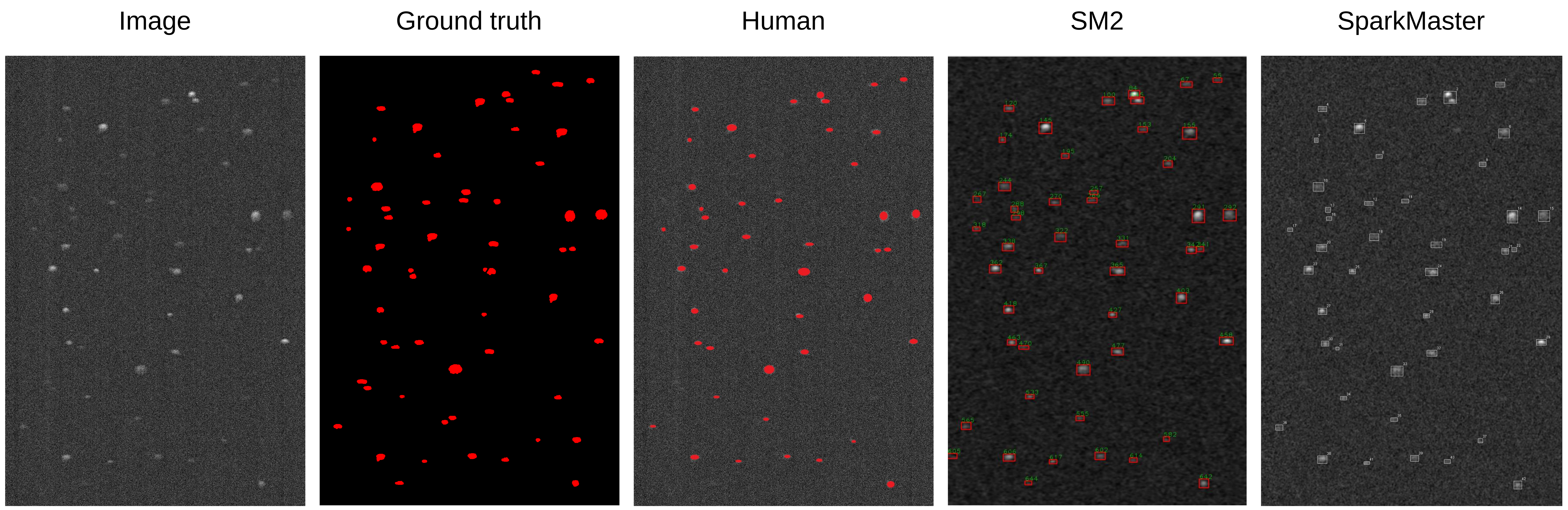
Image
Ground truth
Human
SM2
SparkMaster

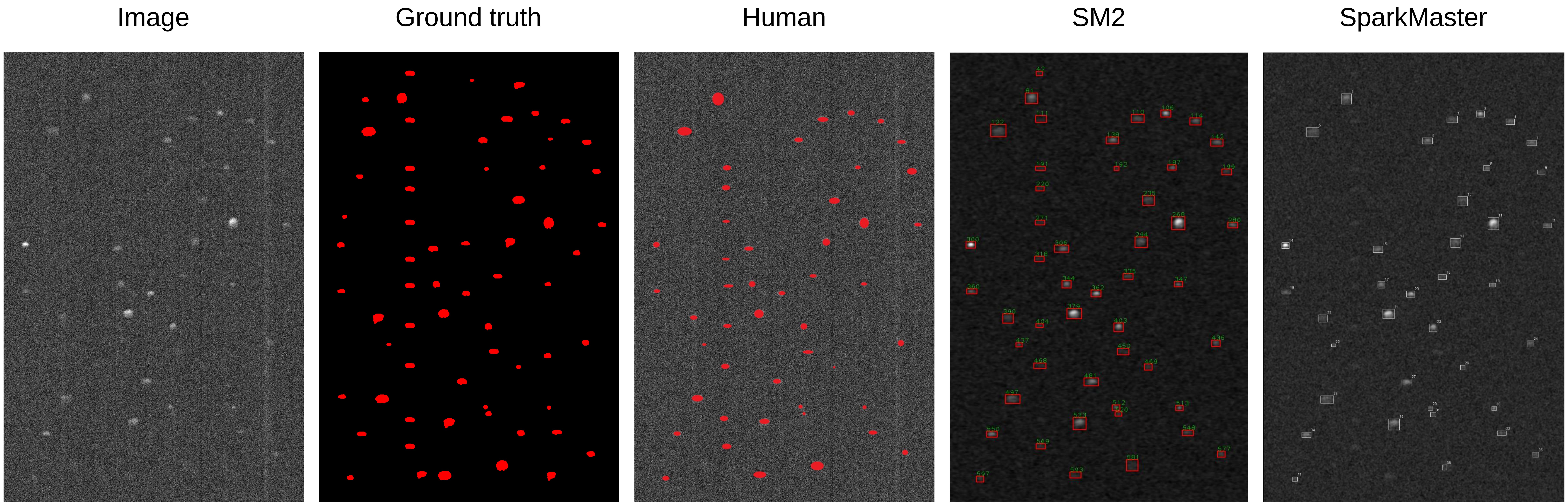
Image
Ground truth
Human
SM2
SparkMaster
