# Supplementary material for: SparkMaster 2: A New Software for Automatic Analysis of Calcium Spark Data
Source: Circ Res. 2023 Aug 9;133(6):450–62. doi: 10.1161/CIRCRESAHA.123.322847 (PMC7615009; doi:10.1161/CIRCRESAHA.123.322847)
Supplement: Supplementary file 2 [file res-133-450-s002.pdf]

We further investigated the relatively weak performance of SparkMaster with the hypothesis that some of the false positives and/or negatives may be related to a mere poor coverage of the detected sparks. Previous users of SparkMaster noted that it sometimes detects only tiny regions within a genuine calcium spark. Given how our algorithm for quantifying false positive and false negative detections works (see Methods), a very small segmented object within a genuine spark may manifest as both a false positive and a false negative detection. This follows from the fact that such pair of genuine and predicted spark may have a very small overlap as quantified using the Dice coefficient ($2\frac{|X \cap Y|}{|X|+|Y|}$: numerator is the # pixels in the overlap of the true and the predicted object, with the denominator being the sum of pixel counts of the two objects). When the Dice overlap is smaller than a given value (0.15 by default in SM2), the genuine spark is not sufficiently covered by a predicted spark (yielding a false negative), while, in turn, the predicted spark has insufficient overlap with a ground-truth spark (yielding a false positive). We therefore repeated the analysis with only a minuscule 1% overlap coefficient between a ground-truth and predicted spark being sufficient for a classification of the prediction as correct, shown in the image below. While this reduces the number of false positives and negatives by SparkMaster markedly, SM2's performance is clearly superior. In addition, although the potential poor coverage of sparks by SparkMaster may not be a problem when estimating the spark rate, it is likely to substantially perturb the estimation of spark features. Interestingly, reducing the degree of overlap required had no effect on the detection performance of SM2.

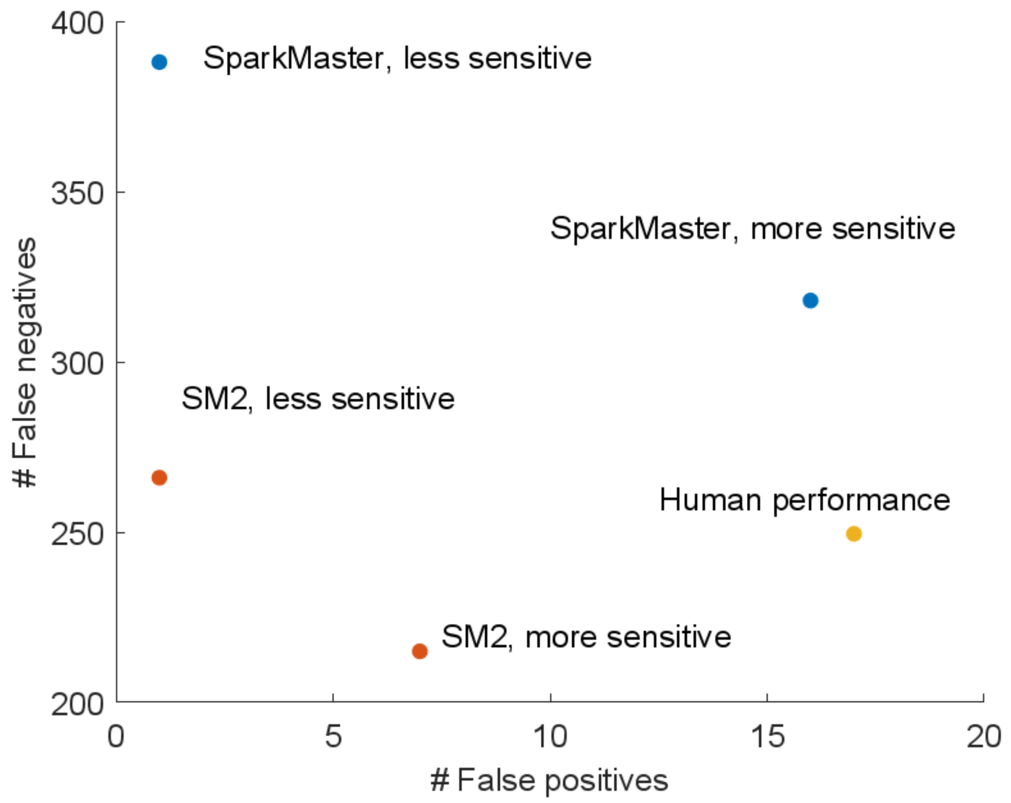

An analogy of Figure 5B in the main manuscript, using 0.01 (rather than 0.15) as the minimum Dice coefficient that is considered a detection of a true spark by a predicted spark.
