# Supplementary material for: SparkMaster 2: A New Software for Automatic Analysis of Calcium Spark Data
Source: Circ Res. 2023 Aug 9;133(6):450–62. doi: 10.1161/CIRCRESAHA.123.322847 (PMC7615009; doi:10.1161/CIRCRESAHA.123.322847)
Supplement: Supplementary file 3 [file res-133-450-s003.pdf]

Supplementary material 3

Demonstration of original SparkMaster performance on Figure 6 data.

Default (3.8) and lower (more sensitive) thresholds are compared.

Using original SparkMaster

Rabbit atrial myocytes

Threshold 3.8

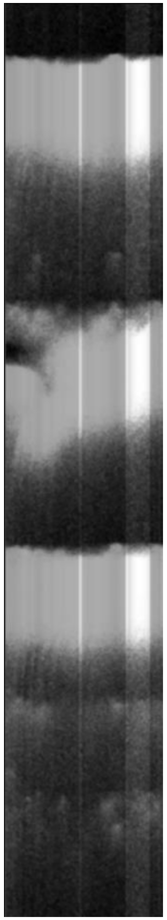

Threshold 2

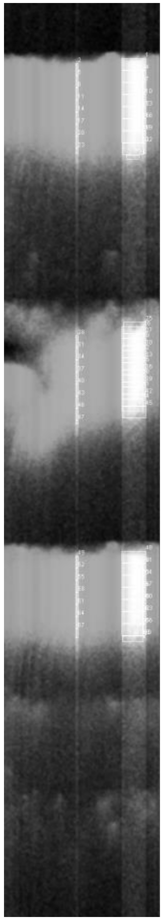

- No sparks detected at default threshold (3.8).
- For the most sensitive threshold possible (2), only oversegmented regions of wave detections are misidentified as sparks.
    - The cause for regions of enhanced brightness (not present in Fig. 6) is unknown.
- At the same time, the sensitivity cannot be further increased, as SparkMaster's threshold value cannot be lowered further.

Vascular smooth muscle cells, Cal-520

Threshold 3.8

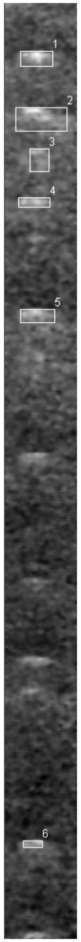

Threshold 2.5

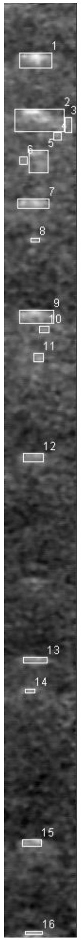

- For the default threshold of 3.8, a number of clear (as well as dim) sparks are missed.
- When the sensitivity is increased, using the threshold of 2.5, most sparks are detected. However, multiple falsely positive detections occur (small sparks numbered 3,4,6 – shown over top right corners).

Vascular smooth muscle cells, Fluo-4

Threshold 3.8

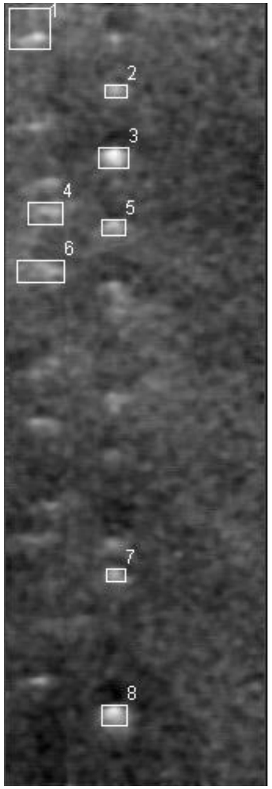

Threshold 2.5

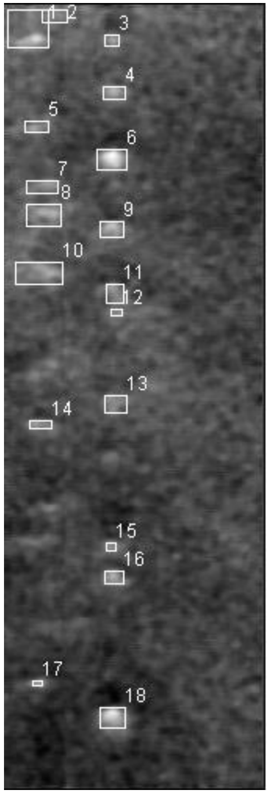

- The default threshold of 3.8 leads to a high number of missed sparks.
- The performance using the threshold of 2.5 appears quite good, although certain relatively clear sparks (e.g., labeled 14, 31 and 34 detected by SM2 in Figure 6) are missed and the spark labeled 2 here is most likely a false positive.

Vascular smooth muscle cells, GCaMP2

Threshold 3.8

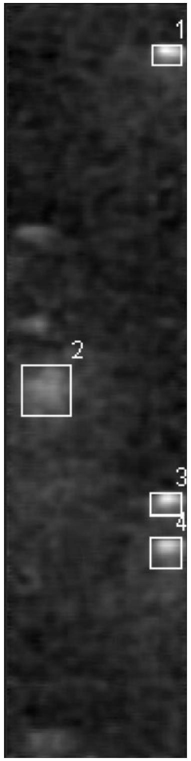

Threshold 2

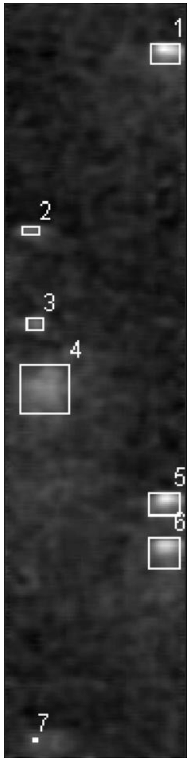

- The default threshold of 3.8 leads to several clear sparks being missed.
- Sparks appear to be correctly detected using the lowest possible threshold of 2, although particularly the bottom one remains poorly covered.
