# Supplementary material for: SparkMaster 2: A New Software for Automatic Analysis of Calcium Spark Data
Source: Circ Res. 2023 Aug 9;133(6):450–62. doi: 10.1161/CIRCRESAHA.123.322847 (PMC7615009; doi:10.1161/CIRCRESAHA.123.322847)
Supplement: Supplementary file 5 [file res-133-450-s005.pdf]

Supplementary Methods

A graphical illustration of the detection of Ca release events by SM2

Parameters:
This box contains a list of parameters in the GUI that are used in the procedure shown at the given slide.

General structure in steps

1. Trimming extracellular space (optional)
2. Column-based image normalization (optional)
3. Row-based image normalization (optional)
4. Image denoising
5. Detection of candidate release events (candidate sparks, waves, …)
6. Identification of long sparks
7. Scoring and detecting normal sparks
8. Scoring and detecting waves and miniwaves
9. Splitting/re-segmenting waves and miniwaves
10. Splitting/re-segmenting ordinary sparks
11. Final visualizations

Parameters:
Basic/Extracellular space threshold determines the fluorescence level under which a pixel is considered to belong to extracellular space (or any other space to be discarded)

Step 1: Trimming extracellular space

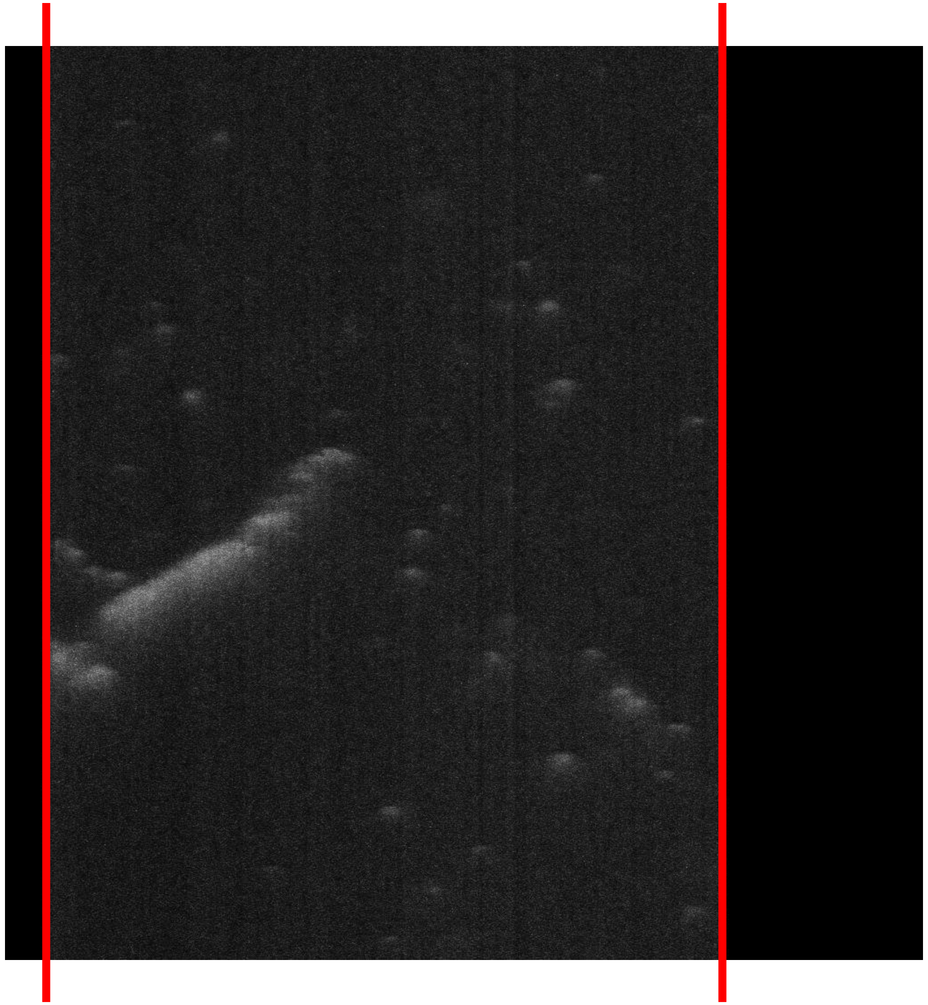

Zones outside the red lines would be discarded

- It may happen that the recording contains dark bands at the sides – this would be typically the extracellular space with little calcium dye.
- SM2 can trim off such areas before further analysis using the basic parameter "Extracellular space threshold".
  - For each column, SM2 detects the proportion of pixels that have intensity below this threshold (10 by default)
  - We trim off the longest possible segment of leading (leftmost) columns so that each of them has at least 75% pixels below the threshold.
  - We also trim off the longest possible segment of trailing (rightmost) columns with the same property.
  - *In this way, we are protected against occasional removal of dark bands in the middle of the cells – only segments at the edges of the image may be removed.*

Parameters:
Advanced/Image preprocessing/Spatial background subtraction determines whether the column-based normalization (i.e., over space) is carried out.

Step 2: Column-based normalization (1)

Goal: remove uneven background brightness that is often present in line-scan recordings

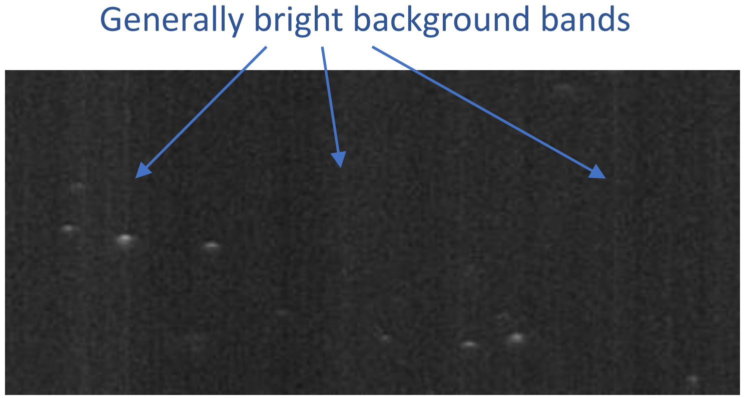

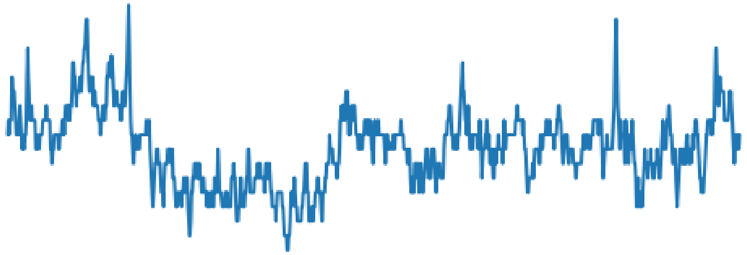

- First, we estimate median background brightness for each column (spot along the line):
  - Calculate the mean and standard deviation of the numbers in the column.
  - Take numbers in the column that are below mean + 1.75 standard deviation (below red line to the right). *This should omit most of Ca spark signal which is brighter.*
  - Calculate the mean and standard deviation of the remaining numbers and keep again only below mean + 1.75 standard deviation (below the yellow line). *While the previous step left out most of spark signal, a part was left – and most of this remainder is discarded in this step, leaving mostly “background-like” pixels. The original SparkMaster does not repeat this step, which is why its sensitivity depends more strongly on the number of sparks and waves in the data, as will be explained in slides later.*
  - Calculate the median of the remaining numbers.
- Doing this for all the columns, we get median background intensity such as shown to the left:

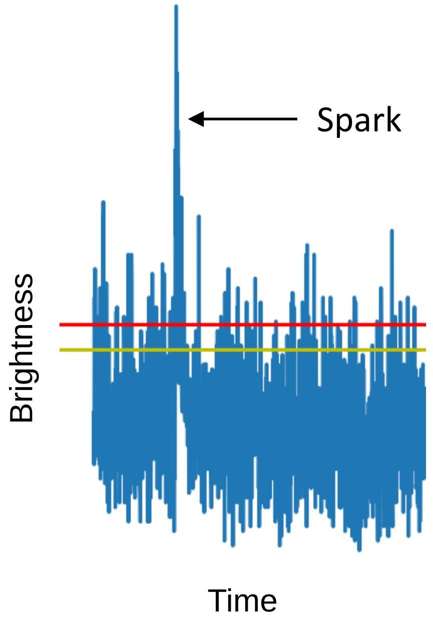

Step 2: Column-based normalization (2)

Dividing each image column by the corresponding median background intensity does the following (note more uniform background):

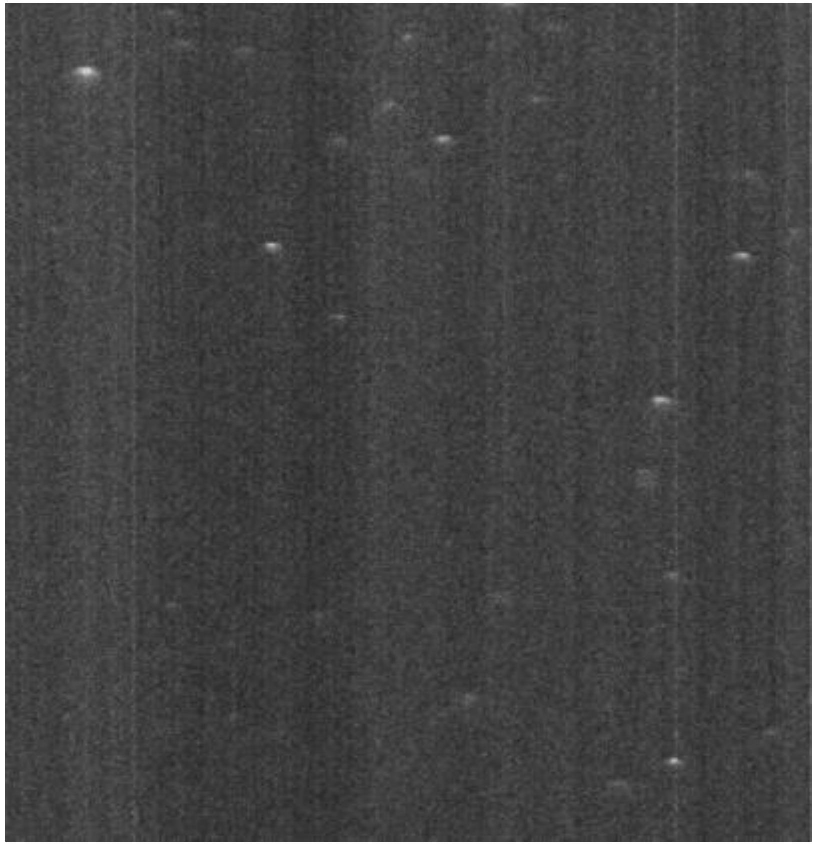

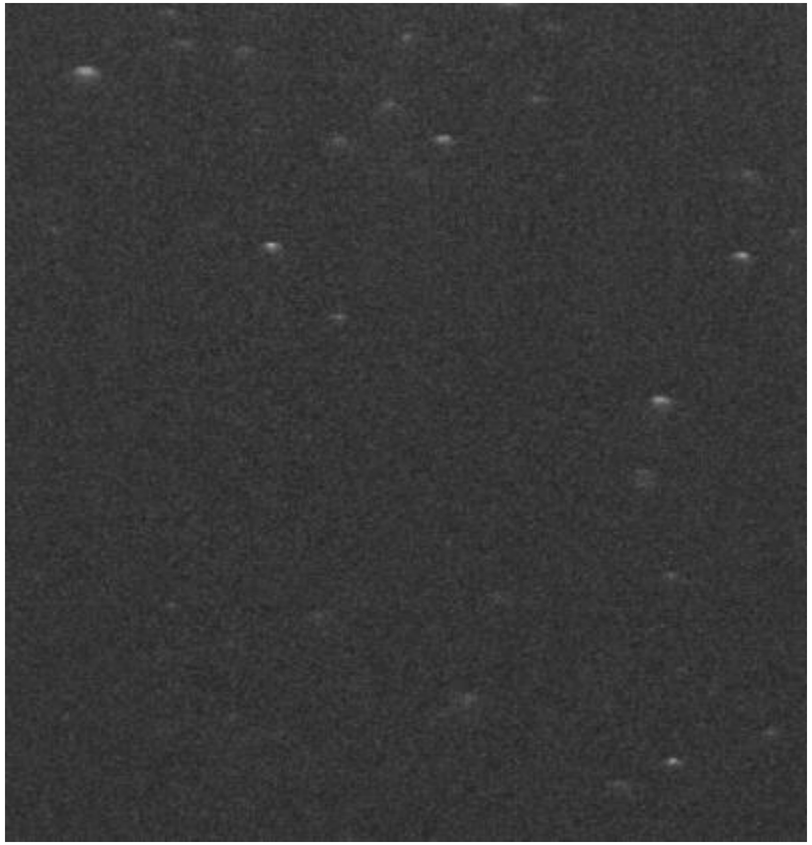

Parameters:
Advanced/Image preprocessing/Temporal background subtraction determines whether the row-based normalization (i.e., over time) is carried out.

Step 3: Row-based normalization

Goal: Remove slow temporal trends from the data.

- The procedure is the same as for columns, except we compute the median for each row (blue curves).
- Subsequently, the resulting curve of median over time is smoothed using a long-lasting median filter, followed by simple averaging (yielding the orange curve).
- At the end, each row is divided by the corresponding element of the smoothed curve to remove temporal trends from the data.

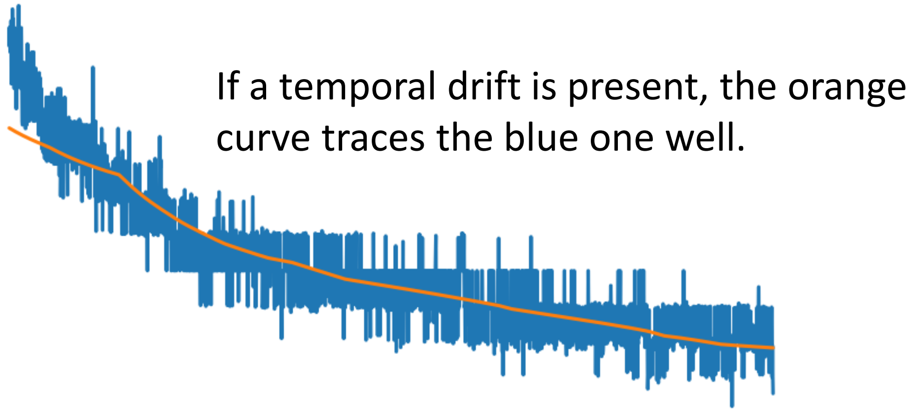

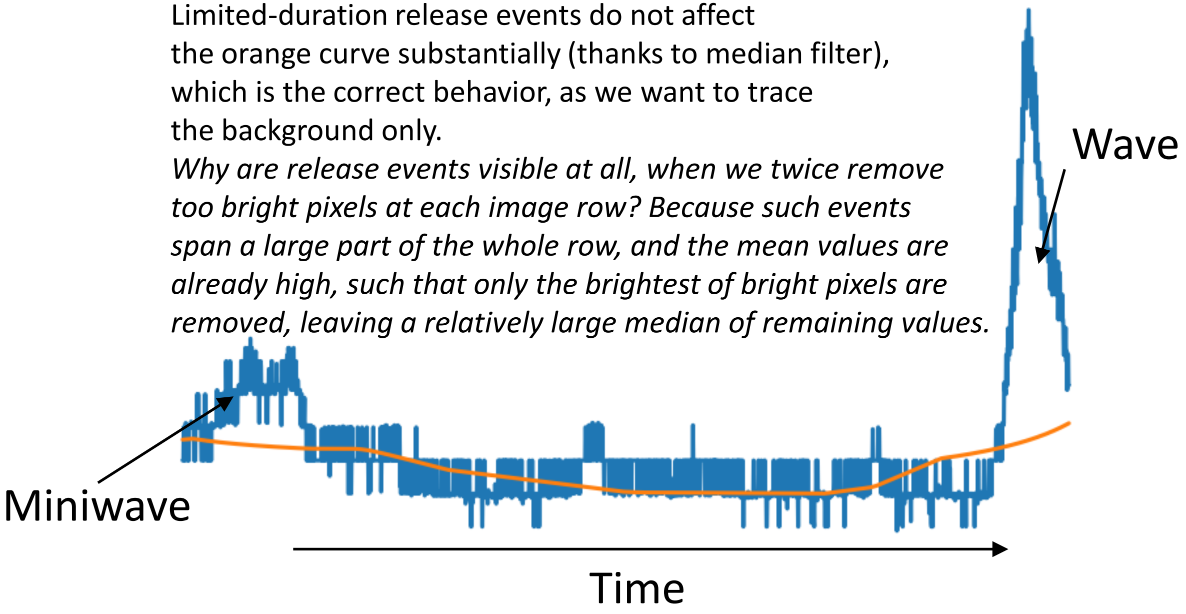

Parameters:
Advanced/Image preprocessing/2D median filtering width, Advanced/Image preprocessing/2D median filtering duration, control the width (#columns) and duration (#rows) of the median filter
Advanced/Image preprocessing/1D Gauss filter width
Determines the filter width of the Gaussian filter (# columns)

Step 4: Image denoising

Normalized image

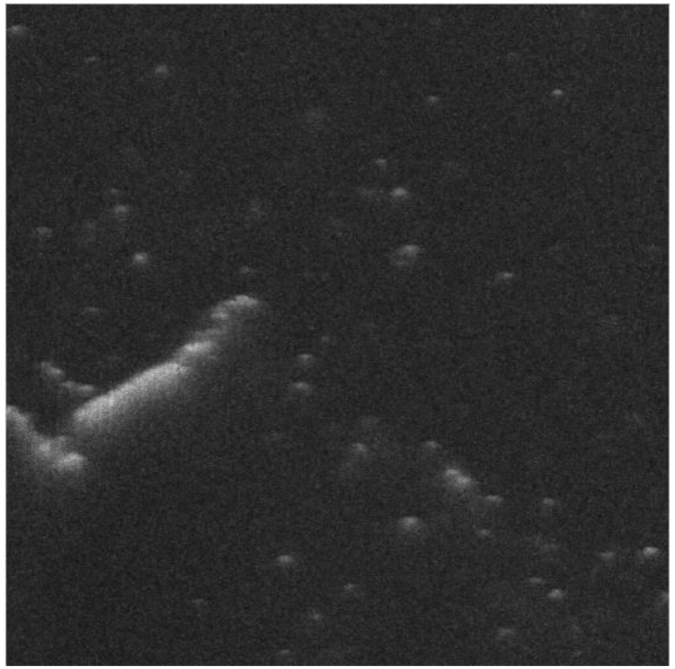

After 2D median filtering

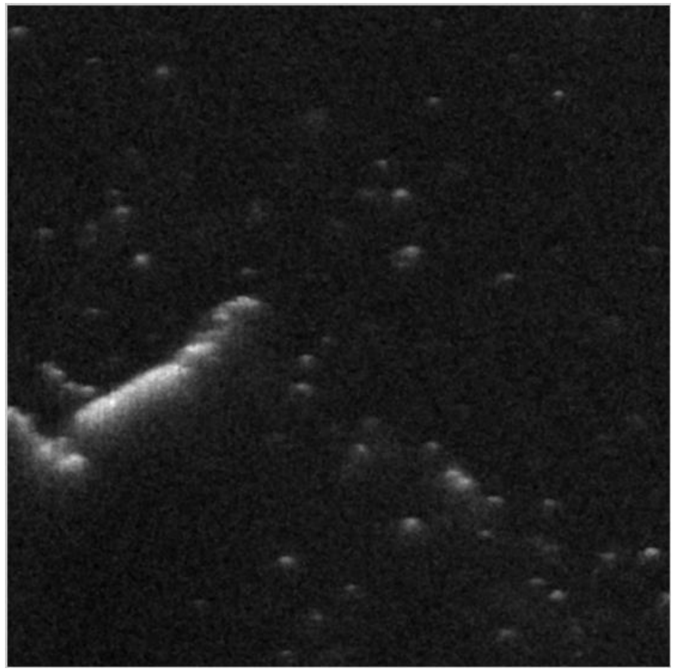

Standard 2D median filtering, with default size of filtering [0.5 µm, 10 ms]; this may be changed by the user.
Median filter is advantageous as it generally preserves object edges well.

After 1D Gaussian filtering

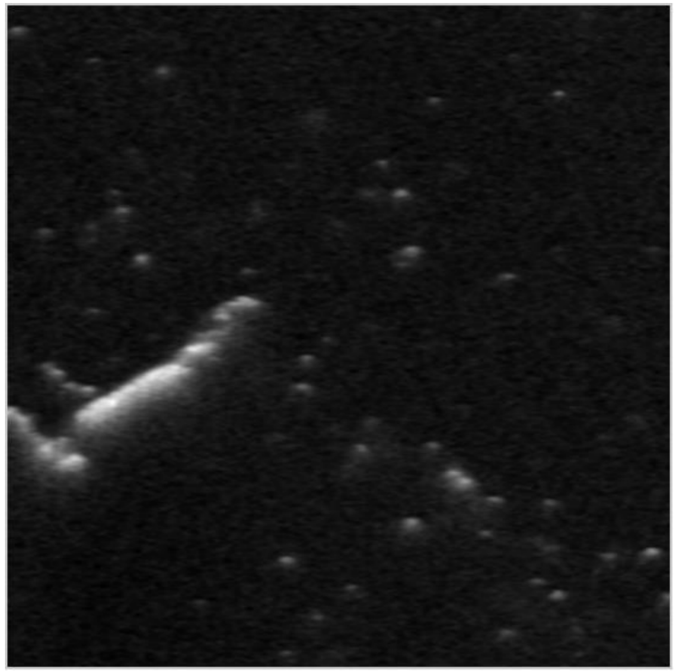

1D Gaussian filtering is applied as a step to reduce noise even further. A pixel is replaced by a weighted sum of nearby pixels <u>on the same row</u>, thus using only spatial information (in contrast with 2D Gaussian filtering, which would use a square region).
In this way, temporal information is not lost/blurred, allowing more precise extraction of spark features.

(Side-note: we also extract a separate copy of the raw image following 2D median and 1D Gaussian filtering like above but not employing the row and column normalization beforehand. This is used later, <u>only</u> when extracting features of detected sparks (using spark locations detected using the normalized images). Using the non-normalized images for measuring spark properties was done so that spark amplitude can be expressed in fluorescence units and at least to some extent compared between recordings taken in similar conditions. This would not be possible when using normalized images.)

Step 5: Detection of candidate release events (1)

Goal: find objects (bright blobs) that have the potential to be sparks or larger release events.

- First step is the estimation of background mean and standard deviation:

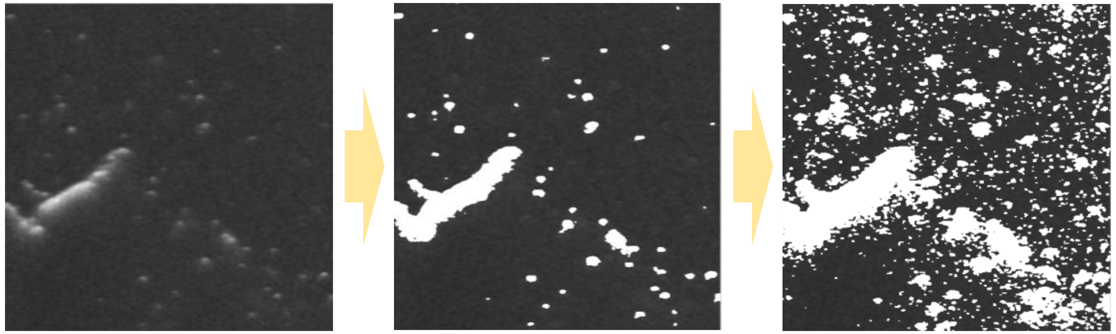

Remove pixels larger than mean + 1 standard deviation (shown in white, these are set to np.nan)

Repeat, calculate the mean and standard deviation of the remaining pixels corresponding to background (shown in black).

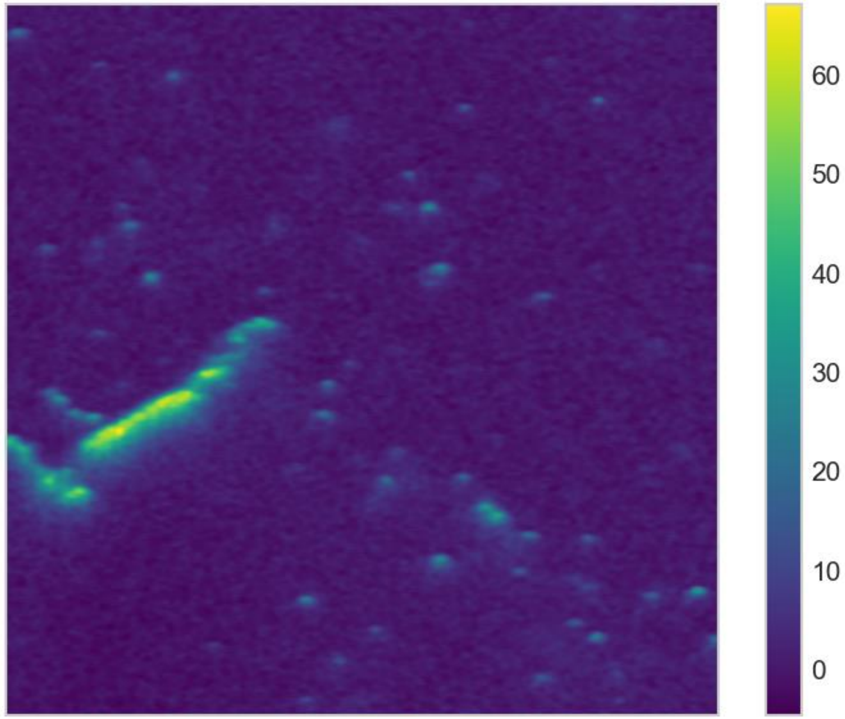

A so-called "SD-transformed" image is created, where the value of a point [i,j] is set to:

$$\frac{img\_denoised[i,j] - mean(background)}{std(background)}$$

where img_denoised[i,j] is the corresponding pixel value in the denoised image. Mean and std of background are corresponding statistics of background pixels (those shown as black in the image to the left). I.e., this image says how many StdDevs of background a pixel is away from the background mean.

Step 5: Detection of candidate release events (2)

Parameters:
Advanced/Spark detection/Object detection threshold is the threshold at which the SD-transformed image is cut to produce the binary mask shown in the middle below.
Advanced/Spark detection/Morphological opening radius is the radius of disk structural element used to perform the morphological opening.

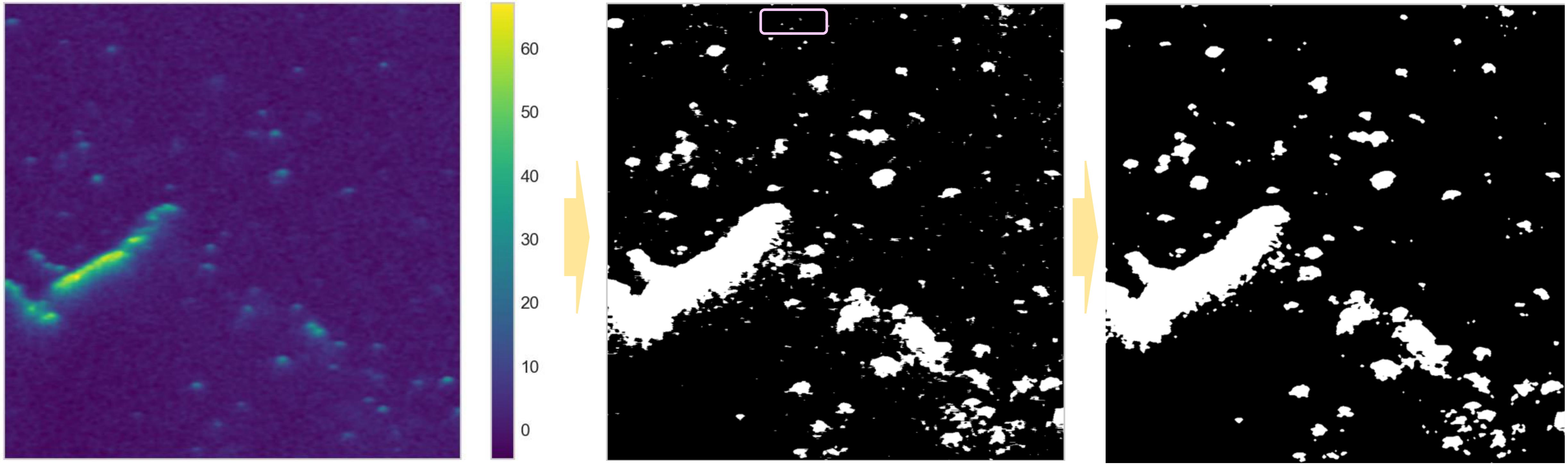

The SD-transformed image is thresholded at a user-specified level (default is 2.75).

And morphological opening is performed to discard small and unpromising objects (with the box showing examples of objects that are removed). Each remaining object (connected component in the binary image) is a candidate calcium release event, and is assigned a unique number and will be processed in further steps.

Comment 1: *The thresholding at a given threshold is more or less where the original SparkMaster stops when detecting sparks. In order to not detect too many false positive detections, it has to use a substantially higher threshold of 3.8 than we can use here, making it miss some genuine sparks. We take an approach where we use a lower threshold, picking up a lot of objects, using further steps to weed out those that are unlikely to be genuine sparks.*

Comment 2: *When the image contains many Ca sparks and waves, the detection of background mean and standard deviation tends to consider some pixels near waves/sparks (their "corona"), contributing higher values to the pool of background pixels. In such recordings, the estimated background standard deviation is thus relatively larger than in spark-sparse recordings, making the overall amplitude of the SD-transformed image smaller. This makes sparks slightly harder to detect. This issue is largely, but not entirely ameliorated by the repeated discarding of foreground (spark-like) pixels when estimating background parameters, shown in the previous slide. SparkMaster does not repeat this, which is why it is much more sensitive to this issue than SM2.*

Step 6: Identification of long sparks

Parameters:
Advanced/Long sparks/Search for long sparks
Long spark detection is performed only if this is ticked.
Advanced/Long sparks/Minimum long spark duration
Any object lasting shorter than this is skipped, as it cannot be a long spark.
Advanced/Long sparks/Maximum long spark width
If the width of one generally bright segment from step 2 is below this, it may be considered further as a long spark.
Advanced/Long sparks/Long spark core diameter
How wide the spark core in step 3 is.
Advanced/Long sparks/Minimum long sparkiness
Minimum long sparkiness for an object to be considered a long spark (otherwise, it is not separated and is treated as a spark further)

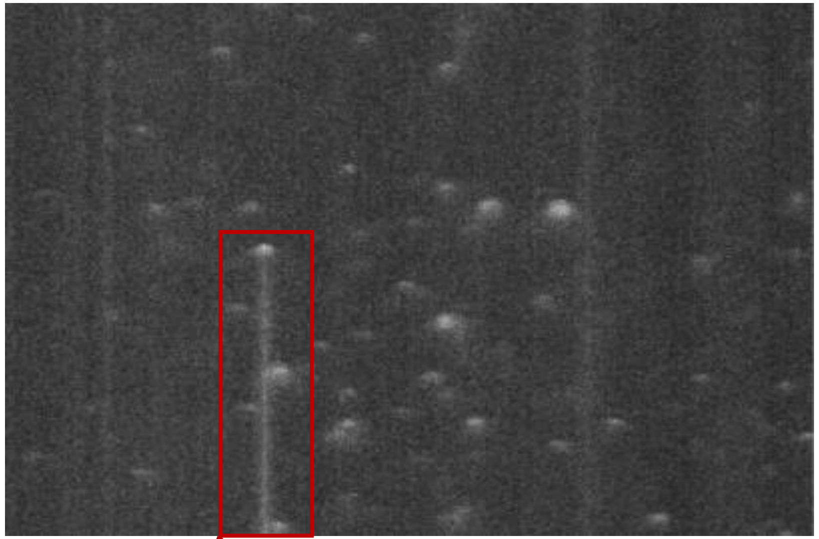

Zoomed-in long spark subimage

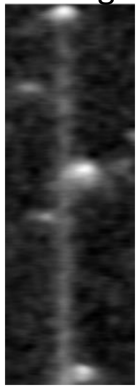

Binary mask of the corresponding candidate object

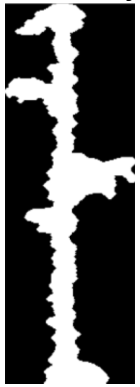

Final SM2 segmentation

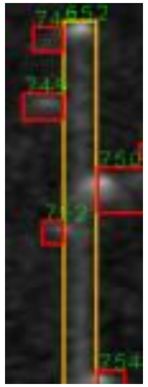

Goal: find long-release sparks.

1. Each candidate release event is first checked for duration – if not long-enough, it is not a long spark.
2. Columns likely corresponding to a long spark center are detected (*the criterion used here is that 25-percentile of the column is at least 2 in the SD-transformed image.*). If there is only one such segment and it is not too wide, the code continues, otherwise the object is considered not a long spark.
3. A central long spark "core" of a given width is considered (shown in red to the right) and "long sparkiness" criterion is calculated. This is defined as the fraction of rows in the given subimage for which the maximum is found within the long spark core.
4. If the long sparkiness exceeds a given threshold, the object is considered a long spark. The part of the binary mask within this core keeps its original number and its status as long spark is recorded. This may leave a number of newly formed isolated objects (generally other sparks occurring near the long spark, shown by arrows to the right), which are assigned new numbers and become candidate sparks. *This criterion serves to discern genuine long sparks from mere long-lasting clusters of Ca sparks chained together, forming a long connected object.*

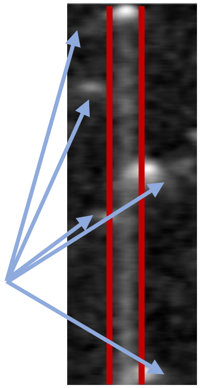

Step 7: scoring and detecting sparks

Parameters:
Advanced/Scoring functions/…
This tab contains parameters of the spark size and brightness scoring functions (midpoint and slope of the sigmoids).
Basic/Spark detection threshold
Objects with compound score at least this high are considered sparks.

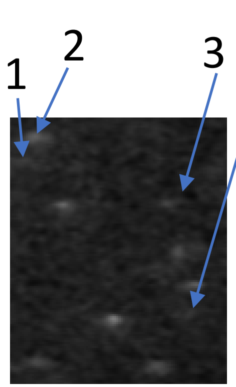

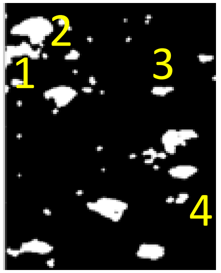

Each candidate event (each white connected component above) gets scored for size and brightness. Concrete scores for 4 release events above are given in the table below.

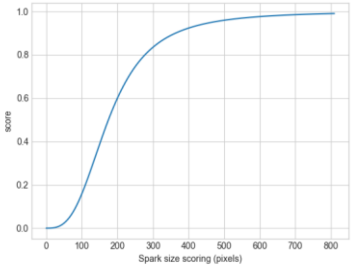

For each candidate release event:

a) Calculate the number of pixels and get the resulting score from the size scoring sigmoid.
b) Calculate the 75-quantile of brightness, subtract the object detection threshold, and find the resulting value on the brightness scoring sigmoid.

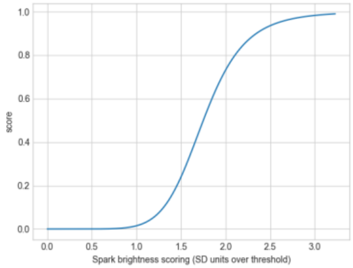

Cell no.	Brightness Q75 over threshold	Brightness score	Size (# pixels)	Size score
1	1.77	0.521	770	0.99
2	4.47	0.999	1220	0.997
3	2.63	0.955	260	0.756
4	1.46	0.208	150	0.401

Size score mask
(color-coded event size)

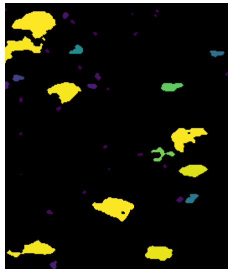

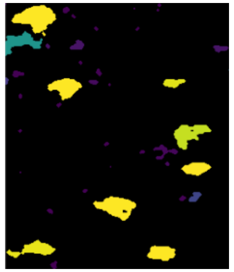

Brightness score mask
(color-coded event size)

The size and brightness scores for each candidate event are multiplied, producing the **compound score**, shown below using color-coding for candidate events.

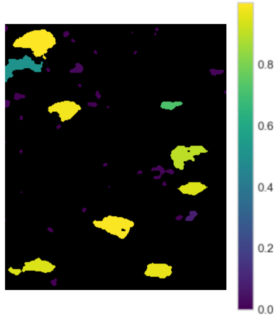

Objects with compound score over a given threshold are then considered real release events (sparks, etc.).

Rationale for the chosen approach: *Using a product of size and brightness score allows detection of all the following types of sparks: 1) clear sparks (both scores high), 2) dim/off-plane sparks (high size score, at least medium brightness score), or 3) small clear sparks (high brightness score, at least medium size score). On the other hand, noise-based candidate objects tend to be ignored, as they produce relatively small and relatively dim objects, yielding low compound score.*
In the example sparks, the obvious spark no. 2 scores well in both measures, whereas, e.g. dim spark no. 1 scores well in size, but not that highly in brightness. Conversely, small spark no. 3 scores well in brightness, but not highly in size. Whether these two would be considered sparks or not will depend on the user's choice of threshold (they would qualify in the default setting). The object no. 4 is either noise or extremely unclear spark, and it scores fairly low in both size and brightness, yielding a very low compound score, meaning it will be typically rejected and not processed further.

Step 8: scoring and detecting waves/miniwaves

Parameters:
Advanced/Scoring functions/…
This tab contains parameters of the spark size and brightness scoring functions for waves/miniwaves (in bottom two rows of the tab).
Advanced/Wave classification/Wave score threshold
Objects with wave compound score at least this high are considered waves.
Advanced/Wave classification/Miniwave score threshold
Objects with wave compound score at least this high are considered miniwaves.

- Separate sigmoid functions are used to score objects with regards to these being larger Carelease events, such as Cawaves or miniwaves. In general, the sigmoid midpoints for the brightness and size are much higher than for sparks.
- If the object's "wave scoring" is high enough, it is labelled a wave, if it is intermediate, it is a miniwave, and if it is low, the object remains a spark.
- Long sparks are excluded from being labelled a miniwave or a wave, even if they score high enough.

Parameters:
Advanced/Wave classification/Wave subcore threshold
The level at which the SD-transformed image (from step 5) is thresholded to produce separate objects, ideally corresponding to the underlying distinct release events.

Step 9: splitting waves/miniwaves (1)

Goal: Separate clusters of calcium waves, miniwaves, and spark clusters (*spark clusters will be sub-split into sparks in the next step*).

General idea: Use high-threshold thresholding to detect distinct cores of smaller release events in potential wave/miniwave clusters.

SD-transformed image

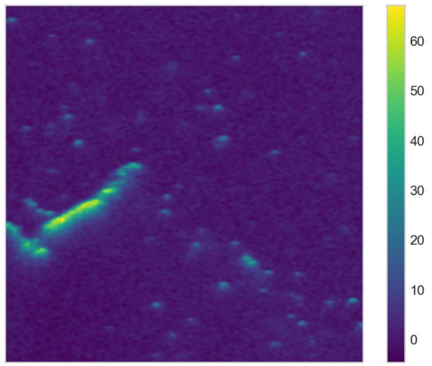

Standard thresholding to find candidate events

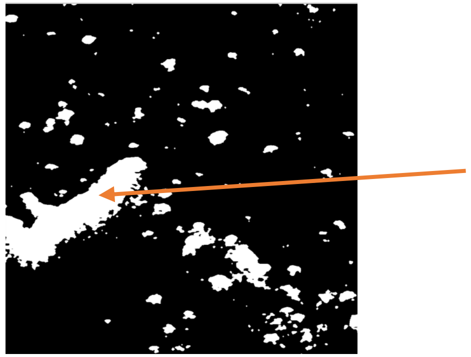

When the image is thresholded at the standard level to find candidate release events, large clusters of release events may be segmented as a single object.

After higher thresholding

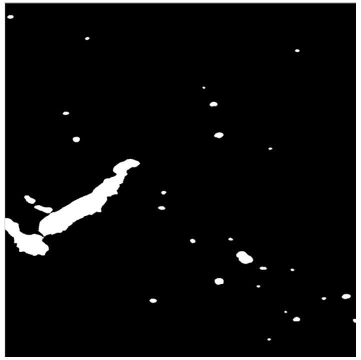

However, when the SD-transformed image is thresholded using a higher threshold, the cluster of release events tends to “fall apart” as shown here. This fact is used in the wave splitting procedure.

Parameters:
Advanced/Wave classification/Wave subcore threshold
The level at which the SD-transformed image (from step 5) is thresholded to produce separate objects, ideally corresponding to the underlying distinct release events.

Step 9: splitting waves/miniwaves (2)

Standard threshold

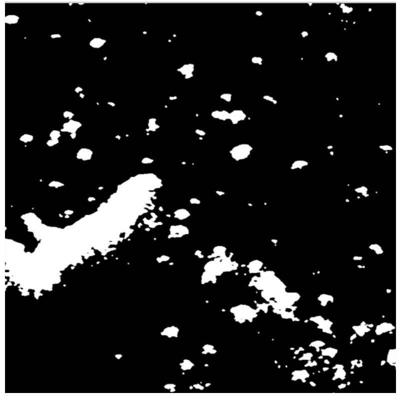

High threshold

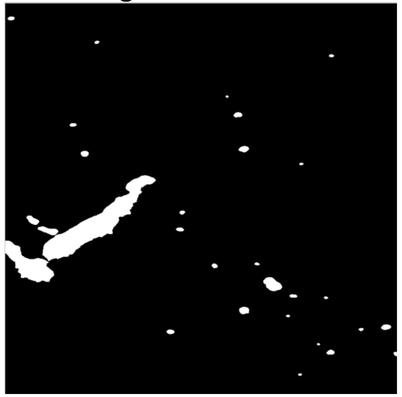

Wave's segmentation visualization **BEFORE** splitting and rejection of low-scoring objects

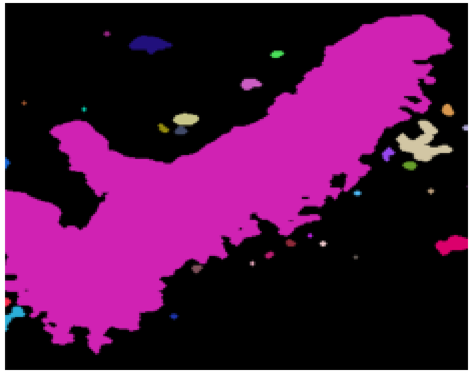

This is a visualization where each connected component in the segmentation mask is given a random color.

Wave's segmentation visualization **AFTER** splitting and rejection of low-scoring objects

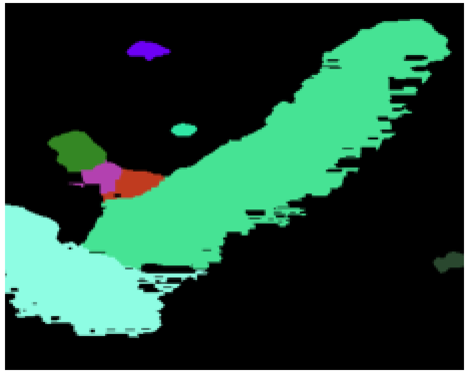

The key point here is how the object originally shown in magenta splits into five distinct parts.

Final bounding boxes for release events (red=spark, blue = wave, cyan = miniwave)

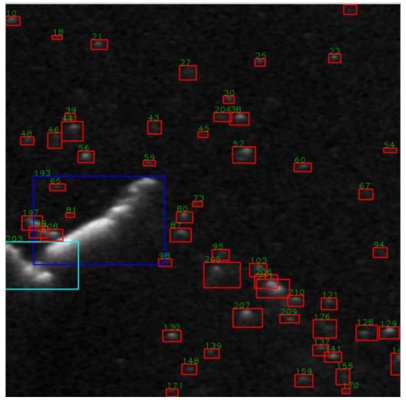

Wave-splitting approach:

For each object scoring high-enough to be considered a wave or a miniwave:

1. Find how many separate objects in the high-threshold mask are present within the standard-threshold mask. If only 1, no splitting is carried out. If more than one, each will correspond to a separate object.
2. For all the separate objects in the high-threshold mask, perform "competitive growing" of the separate objects. This works by gradual expansion of the boundaries of the separate objects in the following way:
3. Cycling over all the objects while any of the boundaries is still expandable, we try to expand the boundary by 1 pixel to all sides from each point on the object's boundary so that:
   a) the pixel that we expand belongs to a candidate release event in the standard-threshold mask.
   b) the pixel does not belong to another object already *(the expansion cannot "capture" other object's pixels)*.
   c) the pixel we expand to does not have a greater brightness than the pixel from which we are expanding.

In this way, we the final extent of objects after splitting "covers" the original mask well. If we used only the high-threshold objects as the final segmentation, they would have a much higher overall brightness and be smaller than other objects detected using the standard threshold. Such an inconsistency makes scoring objects quite challenging and inconsistent, hence we try to "regrow" object boundaries close to the ones given by the standard-threshold-detected object.

By cycling between objects, always expanding at most by one layer of pixels, we that a single growing object cannot dominate the expaascertainnsion. Instead, one gets a typically good and representative coverage of the underlying sparks.

Step 10: splitting spark clusters (1)

Parameters:
Advanced/Spark splitting/Threshold step
Thresholds to be explored are spaced this many SD units apart.
Advanced/Spark splitting/Minimum splitting depth
The minimum required number of consecutive splits required for splitting to be carried out.
Advanced/Spark splitting/Maximum threshold
The maximum threshold that is attempted when splitting a spark cluster. (the lowest threshold attempted is the minimum of the spark cluster's intensity in the SD-transformed image)

Goal: Separate clusters of sparks into single sparks.

A different approach is needed than for waves, given that there is usually no single threshold that works for splitting all the spark clusters in the recording (whereas there tends to be a universal threshold for waves).

General idea: For each potential spark cluster (all candidate release events that are not waves, miniwaves, or long sparks), try splitting it at a range of thresholds. If there is a clear enough cut into multiple sufficiently spark-like object, the cut is performed.

SD-transformed image of a spark couple

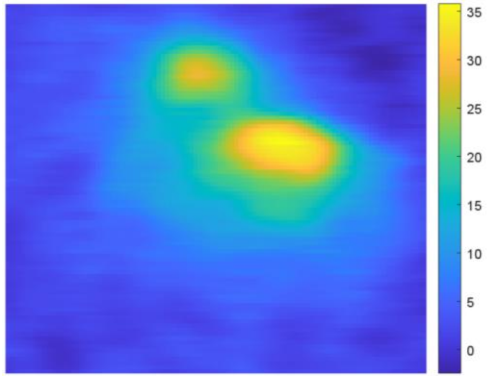

Thresholding is attempted at a range of increasing thresholds, yielding the set of binary masks below:

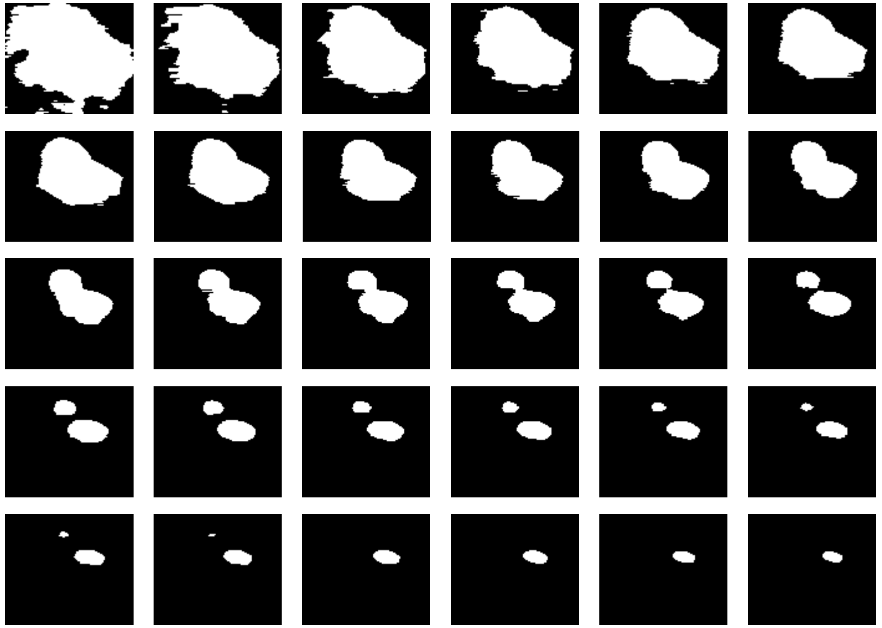

1. For each possible split of the candidate spark cluster, we measure how many sufficiently spark-like objects are formed:
    1. Each separate object in the mask (and the corresponding pixel values of the SD-transformed image) is scored using the standard spark-scoring brightness sigmoid and a "subspark" scoring sigmoid for size (allowing smaller objects to qualify as sparks compared to the spark-scoring sigmoid).
    2. The number of objects with a sufficient compound spark score (same as for normal sparks) is recorded for each threshold.
2. If there is a long-enough segment of thresholds that lead to at least several consecutive splits into more than 1 objects with a sufficient score, a split is performed (at the lowest threshold in the segment. If no such segment is found, no splitting is performed. *For "long-enough", we typically use 2-3. Without requiring several consecutive good splits, we find that sparks can get oversegmented, given that a feasible split can be found by chance from time to time. Conversely, requiring multiple consecutive good splits means that only robustly splittable sparks are indeed split.*
3. When splitting happens, similarly to wave-splitting, objects over the finally selected threshold are competitively "grown back" to cover the original mask of the spark cluster.
4. After performing a split, splitting is recursively attempted in all the newly formed objects. ***Rationale:*** *Imagine a cluster of three near sparks A,B, and C, where sparks A and B can be split using a threshold of 5 and sparks B and C using a threshold of 15. There is no single threshold to split all three of them at once. However, splitting the cluster first at threshold of 5 makes it fall apart into {A} and {B,C}. Splitting of A does not do anything, but the splitting of the object containing B and C detects that a split is possible at the threshold of 15, splitting it into single sparks. Both are recursively attempted to be split, but as they are single sparks, no further splitting is possible and is not carried out. As a result, A, B, and C are now identified as single sparks*

Step 10: splitting spark clusters (2): a trick to speed up spark cluster splitting

Problem: Trying to split all the sparks at a many distinct thresholds (then scoring the subsparks etc.) is one of the more time-consuming parts of SM2 spark segmentation.

Observation: We typically do not need to explore most thresholds in detail, saving time.

Approach taken:

1) When we require at least X consecutive splits into at least 2 spark-like objects, we first process every X-th threshold, counting the objects found
2) Taking thresholds leading to potential splits as seeds, we explore other thresholds nearby (X-1 before and X-1 thresholds after). Example below:

Let's assume we are to explore 26 thresholds and we require at least 3 consecutive good splits to perform splitting. First, thresholds 1, 4, 7, … are explored, yielding 1 or 2 spark-like objects:

Threshold	1	2	3	4	5	6	7	8	9	10	11	12	13	14	15	16	17	18	19	20	21	22	23	24	25	26
# objects	1	?	?	1	?	?	1	?	?	1	?	?	1	?	?	1	?	?	1	?	?	2	?	?	2	?

Pairs of thresholds between 1s do not have to be explored – even if both gave good split into multiple sparks, such segments have no chance of yielding 3 consecutive splits.

These thresholds will be explored, given that they have a chance of producing a segment of length 3 or longer.

How much time do we save? For single sparks (which typically form the vast majority of objects on which splitting is attempted), this reduces the runtime to 1/X, as only every X-th threshold is explored, before it is concluded that no splitting will be performed. The time-saving is obviously slightly smaller for spark clusters that can be split, but remains substantial.

Step 11: Final visualizations (1) Segmentation visualization

Source image

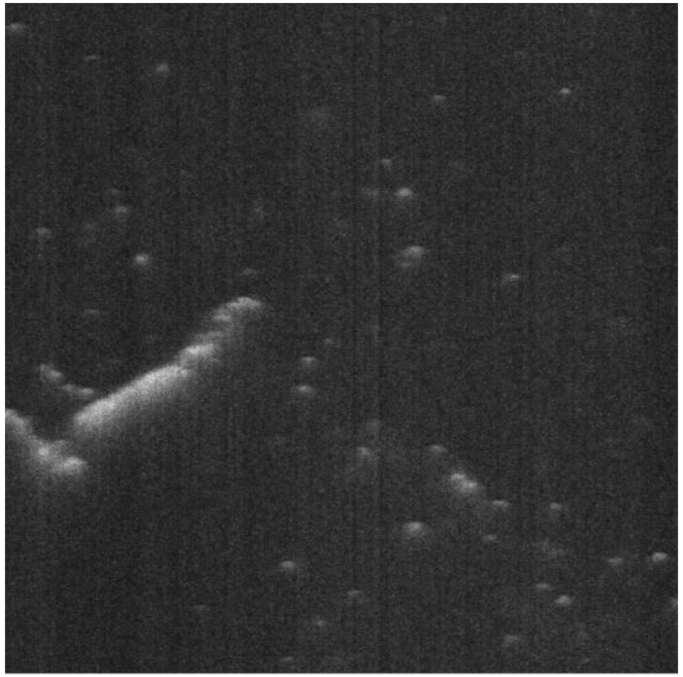

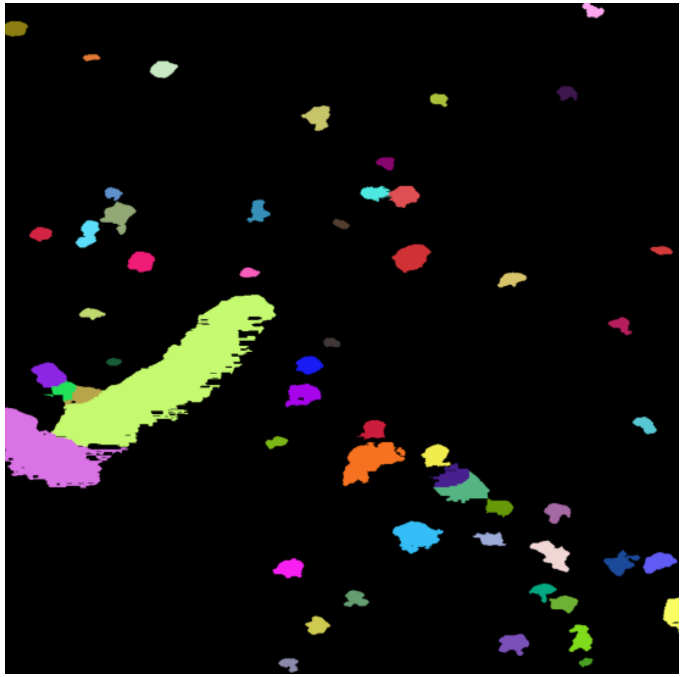

Segmentation may be visualized directly, with each object being shown using a randomly generated color, helping separate distinct objects in Ca release event clusters.

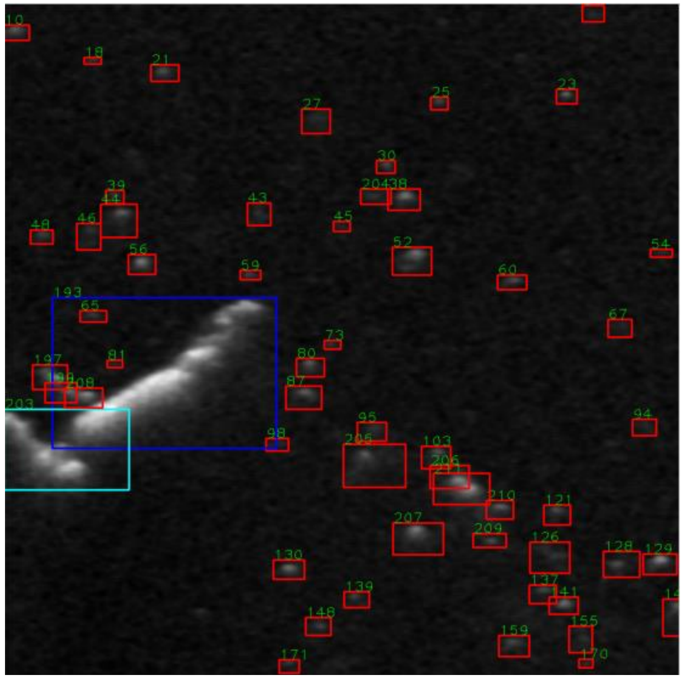

Bounding boxes are alternative visualization, which is shown by default. Distinct colors code for different types of Ca release events.

Other visualizations describing mostly intermediate results from previously described steps may be toggled on optionally in the GUI.

Step 11: Final visualizations (2) (optional) Density maps

Goal: Visualize density of sparks over space-time, aiding identification of sparsely activated regions.

Source Ca release segmentation

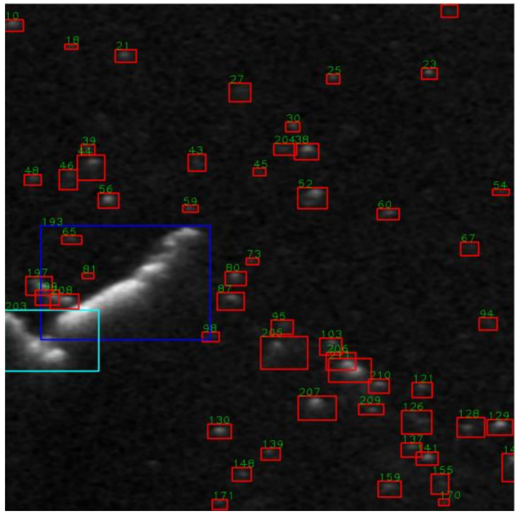

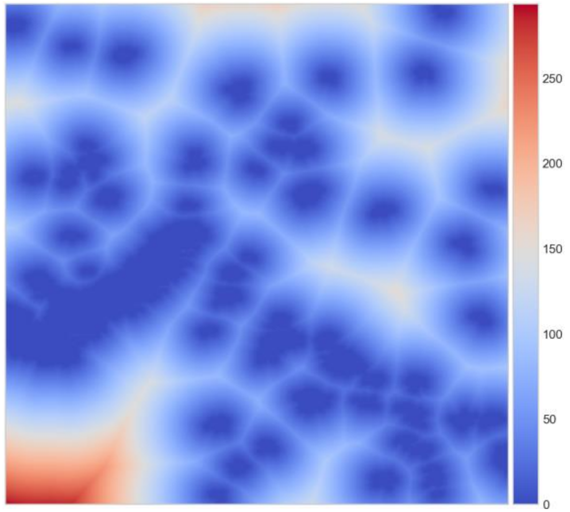

The standard density plot color-codes, for each pixel, how far from the nearest Ca release event the pixel is. The warmer the color, the more distant from Ca release events the pixel is.

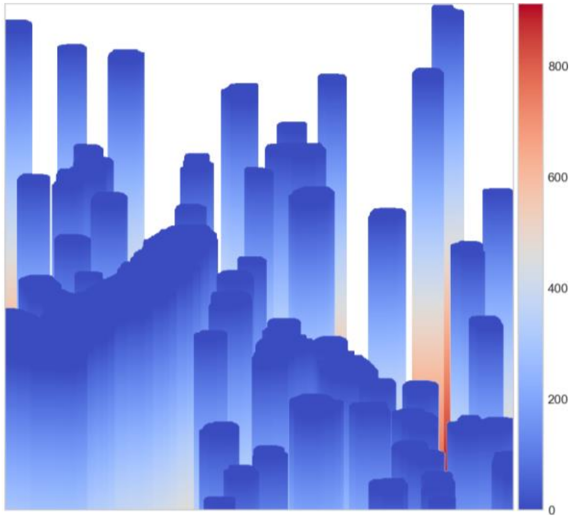

The “skyscraper plot” color-codes, for each pixel, how far the pixel is from the nearest **preceding** Ca release event. This is obtained by going upward from a pixel, measuring the distance to the first row which contains another release event within 2 µm the given column. In this way, one can focus on the distance to release events in a similar location, enabling investigation of SR release refractoriness better than the general density plot might.

Please note that either density plot requires several seconds to calculate, becoming the most time-consuming part of the whole process.
