# Supplementary material for: SparkMaster 2: A New Software for Automatic Analysis of Calcium Spark Data
Source: Circ Res. 2023 Aug 9;133(6):450–62. doi: 10.1161/CIRCRESAHA.123.322847 (PMC7615009; doi:10.1161/CIRCRESAHA.123.322847)
Supplement: Supplementary file 6 [file res-133-450-s006.pdf]

Major Resources Table

In order to allow validation and replication of experiments, all essential research materials listed in the Methods should be included in the Major Resources Table below. Authors are encouraged to use public repositories for protocols, data, code, and other materials and provide persistent identifiers and/or links to repositories when available. Authors may add or delete rows as needed.

Data & Code Availability

Description	Source / Repository	Persistent ID / URL
Source codes and link to compiled software	Github	https://github.com/jtmff/SparkMaster2

DOI [to be added]
